# An approach to quantifying 3D responses of cells to extreme strain

**DOI:** 10.1038/srep19550

**Published:** 2016-02-18

**Authors:** Yuhui Li, Guoyou Huang, Moxiao Li, Lin Wang, Elliot L. Elson, Tian Jian Lu, Guy M. Genin, Feng Xu

**Affiliations:** 1 The Key Laboratory of Biomedical Information Engineering of Ministry of Education, School of Life Science and Technology, Xi’an Jiaotong University, Xi’an 710049, China; 2 Bioinspired Engineering and Biomechanics Center, Xi’an Jiaotong University, Xi’an 710049, China; 3 Department of Biochemistry and Molecular Biophysics, Saint Louis, Missouri 63110, USA; 4 Department of Neurological Surgery, Washington University School of Medicine, Saint Louis, Missouri 63110, USA; 5 Department of Mechanical Engineering and Materials Science, Washington University, Saint Louis, Missouri 63130, USA

## Abstract

The tissues of hollow organs can routinely stretch up to 2.5 times their length. Although significant pathology can arise if relatively large stretches are sustained, the responses of cells are not known at these levels of sustained strain. A key challenge is presenting cells with a realistic and well-defined three-dimensional (3D) culture environment that can sustain such strains. Here, we describe an *in vitro* system called microscale, magnetically-actuated synthetic tissues (micro-MASTs) to quantify these responses for cells within a 3D hydrogel matrix. Cellular strain-threshold and saturation behaviors were observed in hydrogel matrix, including strain-dependent proliferation, spreading, polarization, and differentiation, and matrix adhesion retained at strains sufficient for apoptosis. More broadly, the system shows promise for defining and controlling the effects of mechanical environment upon a broad range of cells.

*In vivo* tissues commonly experience large deformation in both physiological and pathological conditions. For instance, the solid tissues of hollow organs (*e.g.*, bladder) can routinely stretch up to 2.5 times their length, or a nominal strain of 150%^1^. The alveolar can expand to 1.5 times their volume in normal mice and sustainably keep at 3 times in emphysematous mice^2^. Although significant pathology can arise if relatively large stretches are sustained, the responses of cells (*e.g.*, fibroblasts) are not known at these levels of sustained strain^3^,^4^,^5^. The responses of cells to sustained stretches in this upper range has not yet been established definitively for cells in three-dimensional (3D) culture, in part due to challenges with presenting cells a realistic and well-defined culture environment that can sustain such strains^6^,^7^. The need for effective 3D culture systems is particularly pressing because the well-established responses to mechanical stimuli of cells in two-dimensional (2D) culture appear to differ substantially from those of cells in 3D^8^,^9^,^10^,^11^. Even the toolbox of transmembrane molecules such as integrins that cells use to connect with their environment might be different in 3D environments^8^. Further, established tools for mechanobiology in 2D do not extend easily to 3D mechanobiology^12^,^13^,^14^,^15^. Observations acquired using tools such as micropost arrays^16^, 2D traction microscopy^17^, and stretchable substrata^18^ are difficult to relate to 3D due to fundamental differences in the morphologies of cells in 2D and 3D^19^,^20^,^21^.

A number of systems have been developed to probe cell mechanobiology in 3D tissue constructs, but these cannot accommodate the high strains of interest. Although the strains experienced by cells in a tissue stretched 150% can be lower than 150% due to structural features such as crimping^22^ and due to the details of load-sharing between cells and extracellular matrix (ECM)^23^,^24^, existing systems can sustain stretches that are only a small fraction of this, typically on the order of 30%. One of the earliest such systems is slab-like^10^ or ring-like tissue constructs^25^ consisting of cells seeded in a collagen tissue construct. These have provided much insight into issues of tissue remodeling that involve large deformations of the tissue constructs themselves, but only small strains at the level of cells^26^,^27^,^28^,^29^. Like these specimens, tissue constructs synthesized between flexible posts^30^,^31^,^32^ and tissue constructs adhered to flexible substrates^33^ cannot attain the high strain levels of interest. Another attractive 3D system is based on fibrous protein hydrogels, including collagen, gelatin, elastin and fibrin, which exhibit similar structural and mechanical properties with the native ECM^34^,^35^. A variety of fibrous hydrogel systems have been developed to help understand how ECM mechanics regulate cell behaviors^36^,^37^. For instance, alignment of vascular-derived cells was successfully engineered in collagen hydrogels with 10% cyclic strain^33^. Differentiation of cardiac fibroblasts into myofibroblasts was observed in 5% strained collagen hydrogel, which is an important mechanoregulation of fibrosis^38^. However, existing fibrous hydrogel systems can hardly achieve high strain level, which always happens in hollow tissues (*e.g.*, lung and bladder). Another challenge is that these materials systems do not always allow one to perform 3D cell culture where matrix stiffness is decoupled from external mechanical stimulation (*e.g.*, stress/strain). Therefore, it is important to develop a platform that can decouple the effect of matrix stiffness and high strain and help to answer the question how mechanical strain affects cell behaviors.

Herein, we presented defined 3D cellular microenvironments called microscale magnetically actuated synthetic tissues (micro-MASTs) which could undergo reversible, relatively homogeneous deformation up to 160% following non-contact, focused magnetic actuation. μMASTs were stable under sustained, accurate, and large (up to 160%) stretches. We describe here the μMAST system and its application to the study how stretch and ECM stiffness affect proliferation, spreading, polarization, differentiation and apoptosis of several cell types in 3D culture.

## Results

### Optimized μMASTs were stable at high strains

**Figure 1:**
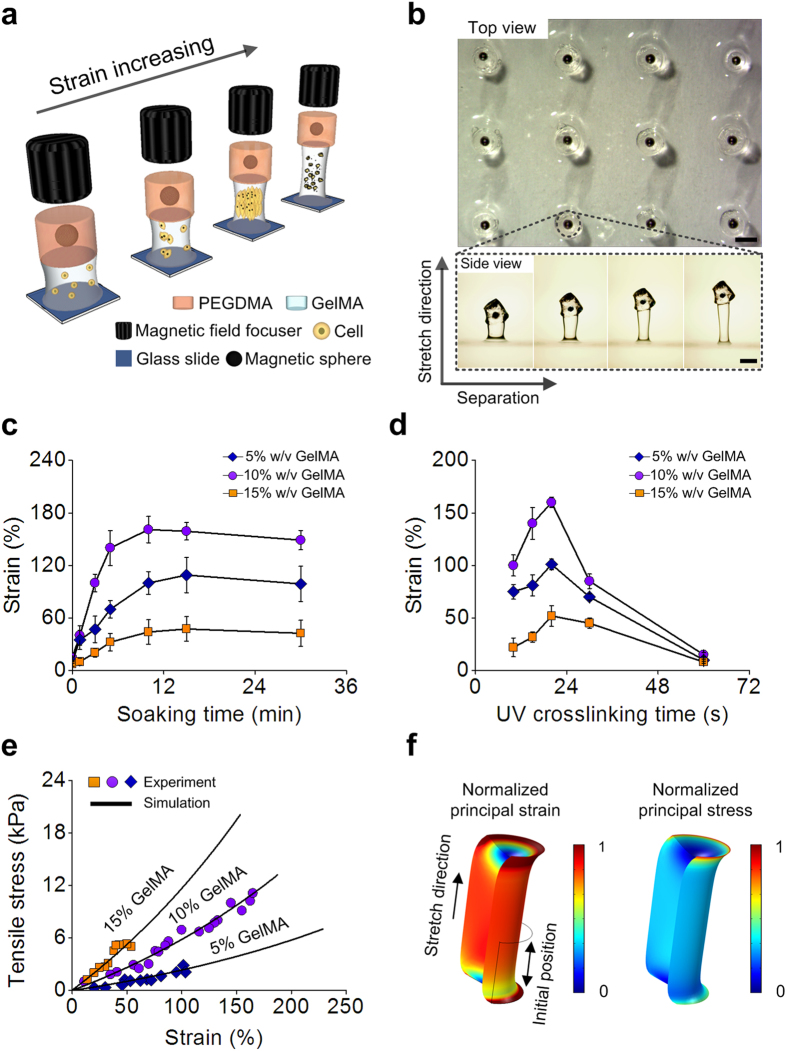
Characterization of microscale, magnetically-actuated synthetic tissues (μMASTs) and interdependence of strain and modulus on cell mechanosensitivity. (**a**) Each μMAST contained a stiff, strong “magnetically-actuated” PEGDMA layer encapsulating an iron microsphere and a tunable-stiffness “synthetic tissue” GelMA layer encapsulating a population of cells. Strains were controlled magnetically using a magnetic field focusing device. (**b**) Top view (upper) of the fabricated μMASTs and side view (bottom) of one stretched μMAST under different straining. Synthetic tissue modulus: 6 kPa. (**c**) The failure strain that μMASTs could withstand prior to failure was maximized by modulating the “soaking time,” the interval between the pouring of the GelMA precursor/cell mixture into the rectangular cut-out in the mold “cover” and the initiation of UV photo-crosslinking. Here, UV photo-crosslinking time was fixed at 20 s and UV light power density was fixed at 2.9 mW cm^−2^. (**d**) UV photo-crosslinking time was modulated to maximize the failure strain. Here, soaking time was fixed at the optimum of 10 min, and UV light power density at 2.9 mW cm^−2^. (**e**) Representative stress-strain curves for synthetic tissue layers composed of different GelMA fraction were highly linear (symbols: experiment; lines: simulation). (**f**) Simulated strain (left) and stress (right) distributions in synthetic tissue layers under magnetic actuation showed highly uniform mechanical fields. Contours were normalized to the peak values. Scale bars: 800 μm.

μMASTs each contained (*i*) a stiff, strong “magnetically-actuated” layer of poly(ethylene glycol) dimethacrylate (PEGDMA) encapsulating an iron microsphere, and (*ii*) a tunable-stiffness “synthetic tissue” hydrogel layer of gelatin methacrylate (GelMA) encapsulating a population of NIH/3T3 cells (Fig. 1a,b, Supplementary Figs S1 and S2). The interface between the two layers was toughened using the double-network principle^39^ (Fig. 1c,d), and differential swelling of the two layers was overcome by tuning the GelMA concentration (Supplementary Fig. S3).

To stretch the synthetic tissues within μMASTs, the iron microspheres were actuated through a magnetic field that was applied focally by a custom-designed array of magnetic field focusers (Supplementary Figs S4 and S5). Each magnetic field focuser was composed of a cylindrical NdFeB rare earth magnet (2 mm in diameter and 2 mm in height) and an iron wire (1 mm in diameter and 8 mm in length). Simulations showed that the magnetic field focusers, held at fixed spacing in a polytetrafluoroethene (PTFE) mold, amplified the field from the NdFeB magnets on the order of tenfold in the direction of the focusing wires to generate a strong and focal magnetic field with minimal crosstalk (Supplementary Fig. S5). When iron microspheres were actuated through focused magnetic fields, μMASTs responded linearly and uniformly up to exceptionally high levels of strain (Fig. 1e,f). The stress-strain curves and ultimate strains were repeatable and tunable by varying GelMA concentration. The focused, high magnetic fields showed no statistically significant effects on cellular responses independent of the mechanical stretches they actuated (Supplementary Fig. S6). μMASTs were synthesized in high-throughput arrays that enabled performance of experiments with high statistical significance.

### Cell responses were dependent upon combined effects of stiffness and strain

**Figure 2:**
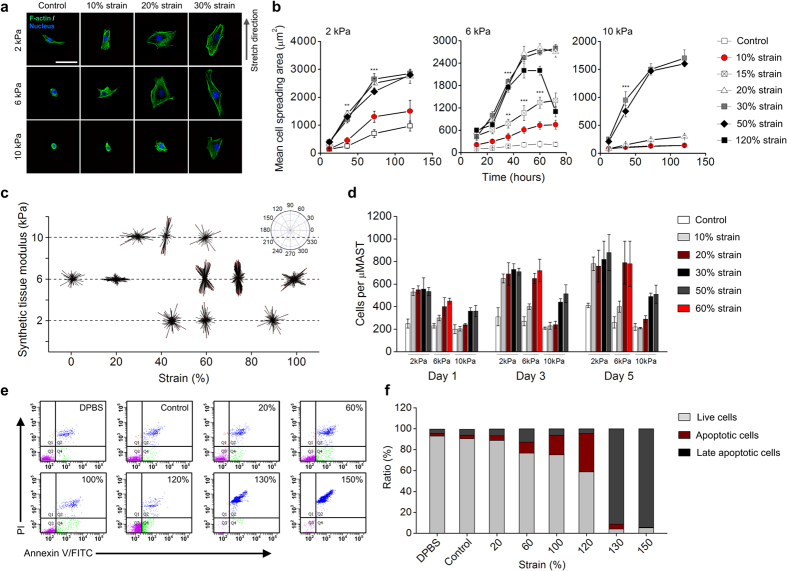
Cellular responses in strained μMASTs. (**a**) Confocal fluorescence images of cells in μMASTs after 3 days of straining to different levels (Green:F-actin (phalloidin); blue: nuclei (DAPI)). (**b**) Mean cell spreading area increased with culture time and with strain level, up to a threshold. In all cases, cell spreading increased nonlinearly with increasing strain, rising rapidly to an asymptotic saturation level at a critical strain in the range of 20–30%. In 10 kPa synthetic tissues, the rise in mean cell spreading area was nearly an order of magnitude, 5 times greater than for the more compliant synthetic tissues. (**c**) Polarization of NIH/3T3 cells in strained μMASTs. For 6 kPa μMASTs, cells oriented perpendicular to the applied stretch at lower strain levels and parallel to the applied stretch (90°) at higher strain levels. At the lowest and highest levels of stretch, no dominant polarization was observed. For 2 and 10 kPa μMASTs, cell orientation with the direction of stretch was evident at intermediate levels of strain. (**d**) The number of cells per μMAST increased with time, and up to a threshold (10% strain for 2 kPa μMASTs, 20% strain for 6 kPa μMASTs and 30% strain for 10 kPa μMASTs, respectively), with different strain levels. (**e,f**) Quantification of live, apoptotic and late apoptotic (dead) cells in μMASTs as a function of straining showed a substantial drop in cell viability in the range of 100–150% strain. Cells in μMASTs were analyzed for apoptosis using Annexin V/FITC and PI staining and flow cytometry after 5 days of straining to prescribed levels. Live cells: Q3 (Annexin V(−)/PI(−)); early apoptotic cells: Q4 (Annexin V(+)/PI(−)); late apoptotic cells: Q2 (Annexin V(+)/PI(+)). Control group: cells cultured in μMASTs without straining. Synthetic tissue modulus: 6 kPa. Error bars, s.d. (**b–d**: 10 ≤ n ≤ 15 μMASTs for each strain level; **e,f**: 60 ≤ n ≤ 80 μMASTs for each strain level, **p < 0.01, ***p < 0.001). Scale bars: 50 μm.

Several cell responses, including cell spreading, polarization and proliferation, relate to important observations in development and in granulation tissue. To clarify whether the μMASTs remain stiff with increasing culturing time, we firstly tested the elastic modulus of μMASTs (6 kPa) at day 1, 3 and 5 of culturing, respectively (Supplementary Fig. S7). The results suggested that there is no significantly decrease even after 5 days culturing, indicating that the proliferated cells and their secreted matrix will not have an obvious effect on remodeling the local microenvironment in μMASTs. The effects of matrix stiffness on spreading were diametrically opposite those observed in 2D culture. NIH/3T3 cells in 2D culture spread more on stiffer substrata^40^; however, the opposite was observed for NIH/3T3 cells in 3D, with cells in the 10 kPa synthetic tissues spreading much less on average than those in the 2 and 6 kPa synthetic tissues (Fig. 2a,b and Supplementary Fig. S8). In all cases, cell spreading increased nonlinearly with increasing strain, rising rapidly to an asymptotic saturation level at a critical strain in the range of 20–30%. In 10 kPa synthetic tissues, the mean cell spreading area increased almost 5 times when strained up to 30%. These phenomena can be explained only through active cellular processes: spreading area was greater that could be explained by passive stretching of cells, and the lower spreading area in stiffer synthetic tissues would not be expected if passive strain simply opened more volume for cells to occupy. The ~72 hour time interval that cell populations required to spread to steady state mean size did not depend upon strain and matrix stiffness. A possible reason for stronger cell activity in softer μMASTs is that μMASTs with lower modulus and monomer concentration may be easier to be degraded by encapsulated cells. It is well-known that matrix metalloproteinase (MMP) is a kind of proteolytic enzymes and can degrade virtually any component of the ECM and remodel their local microenvironment, whose expression and activation is increased in almost all human cancers compared with normal tissue^41^. To clarify if the MMP activity of cells will significantly degrade their local matrix and regulate cell behaviors in μMASTs, we tested the cell spreading and proliferation in strained group immersed in culture medium with a broad-spectrum MMP inhibitor (Batimastat, BB94). The results indicated that BB94 significantly inhibited the activity of cell behaviors, including mean cell spreading area and proliferated cell numbers, in all three kinds of μMASTs (2 kPa, 6 kPa and 10 kPa, respectively) during 5 days culturing (Supplementary Fig. S9). In 50% strained 6 kPa group with BB94, the maximum mean cell spreading area is 1850 ± 200 μm^2^ and the cell numbers per μMAST is 620 ± 120 after 5 days of culturing, which is lower than group without BB94 (mean cell spreading area is 2550 ± 100 μm^2^ and cell numbers per μMAST is 750 ± 50). These phenomena can be also found in 2 kPa and 10 kPa strained groups. However, the increasing trend of mean cell spreading area and proliferated cell numbers keep consistent with the group without BB94, suggesting that mechanical strains still made significant contributions to enhance cell activity in μMASTs.

Polarization of cells and stress fibers were also functions of both strain and modulus. In unstrained synthetic tissues with an elastic modulus of 6 kPa, NIH/3T3 cells showed random orientation (Fig. 2c and Supplementary Fig. S10). However, cells polarized perpendicular to the stretch direction when the synthetic tissues in which they were cultured under 20% strain for 3 days. This response is consistent with cellular responses at the periphery of a wound but inconsistent with observations of cells cultured on flexible 2D substrata, which always align with the direction of a sustained stretch, and cells cultured on isometrically stretched substrate, which orient randomly^42^. The dominant strain-energy theories of polarization predict the latter^43^, suggesting that the principles underlying cell polarization in 3D differ fundamentally from those in 2D. The significance of biphasic alignment for 6 kPa stiffness μMASTs occurred at 60% strain level. To check if the MMP activity affects this behavior, we added MMP inhibitor (BB94) in culture medium of 6 kPa μMASTs with 60% strain (Supplementary Fig. S11). Both fluorescent images and alignment quantification showed that the biphasic alignment disappeared and cell reoriented perpendicular to the strain direction. These results indicated that cell polarization in 6 kPa μMASTs at 60% strain could be dependent on the concentration of ligand and MMP-susceptible sites. Further studies should focus on testing the distribution of adhesion ligands and MMP-susceptible sites under mechanical strain. Cells in 75–90% strained synthetic tissues showed increasing polarization parallel to the stretch direction, but coordinated polarization diminished in synthetic tissues strained above 100%.

The applied strain will affect cell polarization in all three kinds of μMASTs (2 kPa, 6 kPa and 10 kPa). In synthetic tissues with a modulus of 10 kPa, cells orientated perpendicular to the stretch direction at strain levels up to 30%, and parallel to the stretch direction for strains above 40% (Fig. 2c and Supplementary Fig. S10). In synthetic tissues with a modulus of 2 kPa, however, orientation perpendicular to the stretch direction was never observed; cells aligned parallel to the stretch direction with peak parallel alignment at a strain of 60%. These observations are again consistent with variations in cellular orientation seen in granulation tissue, with cells least aligned in the most compliant areas.

Strain-activation was required for substantial proliferation of cells in all μMASTs tested (Fig. 2d and Supplementary Fig. S12). Straining of 10–20% was required, with the threshold increasing with matrix modulus. A saturation value was reached in all cases except at the highest strain levels (>100% in the 6 kPa synthetic tissues), in which proliferation ceased and gave way to apoptosis (Fig. 2e,f and Supplementary Figs S13).

Cellular cytoskeleton is a dynamic, adaptive and functional entity that can connect the cell physically and biochemically to the external microenvironment and generate coordinate force enabling the cell to change shape and polarization^44^. A key bridge to cytoskeleton and ECM is focal adhesions (FAs). For instance, existing studies demonstrated that myosin II and other crosslinkers can generate traction forces on FAs by which they are anchored to ECM surrounding the cell^45^. Theoretical modeling also suggests that the interaction of the contractile force generated by the cellular stress fibers and FAs with the external mechanical strain triggers cell repolarizations^46^. However, most of these studies still stagnate in the interactions between cells and 2D substrata, which can hardly match with *in vivo* situation because of its complexity. Our system provides a potential to move this from 2D to 3D and can be used to study the mechanism that links mechanical force to cellular responses, which is important during tissue development and disease. Further challenges still need to be addressed. For instance, cells in hydrogels may dynamically adhere to matrix during the stretching process. Cell may release their adhesions (*e.g.*, FAs) from the matrix and reattach. There may be no memory of strain when this happens. Therefore, further studies should focus on the dynamic changing of cellular FAs when applied extreme strain on μMASTs.

Another surprise was that cells adhered to the matrix at strain levels sufficient to cause apoptosis, rather than release from it. In 6 kPa synthetic tissues, cell viability decreased gradually from 90% viability at no strain to 70% viability at 100% strain (Fig. 2f). Early apoptosis was evident in the 100% and 120% strain groups, and viability plummeted to 10% at 130% strain, the latter with widespread late apoptosis and cell death. Cells were viable to μMAST failure in the 2 kPa and 10 kPa specimens.

Another key factor that may influence cell behaviors is the change of μMAST’s microarchitectures when applied external strains. To study the deformation of pore structures due to strain alone in three kinds of μMASTs (2 kPa, 6 kPa and 10 kPa), we characterized the pore size, aspect ratio of interconnected pores and porosity of hydrogel samples (Supplementary Figs S14). Our results showed that pore size decreased by increasing the stiffness of μMASTs, which is caused by the increased monomer fraction (Supplementary Figs S14a). For 2 kPa and 6 kPa groups, the average pore size is 180 ± 20 μm and 110 ± 15 μm, respectively, which is larger than 10 kPa group (55 ± 10 μm). Similarly, the porosity of μMASTs decreased with increasing monomer fractions. All these results suggested that the cell activity in μMASTs with larger modulus may be also inhibited due to the less growth space supported by the hydrogel. To clarify the relationship between the hydrogel architecture applied strains, we characterized the aspect ratio of pores in 6 kPa μMASTs under different strain levels (Supplementary Figs S14b-f). The results showed that aspect ratio of pores increased with increasing applied strains and the pore significantly aligned parallel to the stretch direction, which may be a factor to affect cell repolarization in strained μMASTs. The deformation of pore structure may also apply off-axis compressive stresses on encapsulated cells and narrow down the region for cell growth when applied uniaxial strain, which may induce the cell apoptosis. To clarify this, we have compared the area and aspect ratio of cell nucleus in 6 kPa synthetic tissues between 120% and 60% group (Supplementary Fig. S15). The results showed that the nuclear area significantly decreased when applied 120% strain and aspect ratio of cell nuclei is larger than 60% group, indicating that the hydrogel may collapse off-axis and compress the cell, including the nucleus and lead to apoptosis.

## Discussion

The mechanical environment is known to affect cell morphology, proliferation, polarization, and differentiation in both 2D and 3D culture^33^,^47^. The stiffness and strain of the mechanical environment are intertwined in 3D, with fibroblast cells in 3D both stiffening the ECM and proliferating/dying to reach the point at which their modulus match; in cell-seeded collagen ECM, this corresponds to the steric percolation threshold^23^,^48^. Because the stiffness and strain are definable independently with μMASTs, we were able for the first time to probe their effects on cells independently in a 3D culture environment. In addition, μMASTs provided a platform for applying simultaneous, non-contact, and tunable mechanical stimulation to parallel arrays of cells in 3D culture. This platform overcomes challenges faced by other 3D culture systems associated with sample handling, diffusion barriers, specimen variability, and non-uniformity of mechanical loading. The system enabled the first-ever extreme strain testing of cells in a uniform 3D environment.

The μMAST system has limitations that bear mention. For instance, non-fibrous matrix that enabled strain uniformity is not fully representative of the cell environment in a real tissue. Another challenge is that ECM porosity and stiffness cannot be prescribed independently. This presents a challenge to interpreting surprising results about the cellular apoptosis, as we cannot be certain that cells in real tissues will “hold on” at strains associated with apoptosis like those in μMASTs do. However, we believe that μMASTs provide useful and well-calibrated information about cell mechanobiology that can be applied to understand future work in more complicated fibrous mechanical environments. We are also hopeful that the system will continue to be useful for dynamic characterization of mechanobiological responses of cells in 3D.

## Methods

### Preparation and fabrication of μMASTs

We developed a two-step lithography approach to fabricate μMASTs composed of (*i*) a stiff, strong “magnetically-actuated” layer of PEGDMA and (*ii*) a tunable-stiffness “synthetic tissue” layer of cells encapsulated in GelMA. The magnetically actuated PEGDMA layer was created from a poly(methyl methacrylate) (PMMA) mold, and the GelMA synthetic tissue later was created by masked photolithography.

#### Preparation of PMMA mold and magnetic field focusers

A PMMA (Shuguang Plexiglas Co., Ltd.) mold was designed using CAD software (AutoCAD 2010, Autodesk Inc.) and fabricated using a CO_2_ laser engraving system (Universal VLS2.30, Universal Laser Systems, Inc.). The rectangular PMMA mold was composed of three parts, each 50 mm × 36 mm (Supplementary Fig.S16): (*i*) a 1 mm thick cover containing a 20 mm × 24 mm rectangular cut-out; (*ii*) a 1 mm thick interlayer containing 15 circular holes of 1 mm diameter, situated beneath the cover’s rectangular cut-out; and (*iii*) a 2 mm thick base plate.

A custom-designed array of magnetic field focusers was used for both positioning of iron microspheres during fabrication of the magnetically-actuated PEGDMA layers and for mechanical straining of μMASTs. This array was constructed by assembling 15 magnetic field focusing elements on a PTFE (DuPont^TM^, Inc.) base (Supplementary Fig. S5a). Each magnetic field focusing element was composed of a cylindrical NdFeB rare earth magnet (2 mm in diameter and 2 mm in height) (K&J Magnetics) and an iron wire (1 mm in diameter and 8 mm in length).

#### Preparation of GelMA and cells

GelMA, a biocompatible and photocrosslinkable hydrogel that is effective for 3D cell encapsulation, was synthesized as described previously^47^. Briefly, type A porcine skin gelatin powder (Sigma-Aldrich) was added into Dulbecco’s phosphate buffered saline (DPBS; Gibco BRL Life Technologies, Inc.) at a concentration of 10% (w/v) and a temperature of 65 °C, and stirred until fully dissolved. Methacrylate (Sigma-Aldrich) was added into this solution at a rate of 0.5 mL min^–1^ under stirring at 50 °C until the target volume was reached. The solution was then allowed to react for 2 hours. The fraction of lysine groups that reacted could be controlled by varying the amount of added methacrylate. Following a 5× dilution with additional warm (40 °C) DPBS to stop the reaction, the mixture was dialyzed against distilled water using 12–14 kDa cutoff dialysis tubing (Spectrum Labs, Inc.) at 40 °C for 1 week. Water was changed every day to removed salts and methacrylic acid. After that, the GelMA solution was lyophilized for 1 week to generate a porous foam and stored at –80 °C for further use.

NIH/3T3 fibroblasts (Cell Bank of the Chinese Academy of Sciences) were cultured in Dulbecco’s modified Eagle’s medium (DMEM; high glucose, Gibco BRL Life Technologies, Inc.) supplemented with 10% fetal bovine serum (FBS; Gibco BRL Life Technologies, Inc.) and 1% penicillin–streptomycin mixture (Gibco BRL Life Technologies, Inc.) at 37 °C, 95% humidity, and 5% CO_2_. Cells were passaged approximately 2 times per week with the media changed every 2 days.

#### Fabrication of μMASTs

A two-step lithography approach was developed to fabricate μMASTs (Supplementary Fig. S1). In the first step, one iron microsphere (350 μm diameter, nickel-coated) (K&J Magnetics) was placed in each of the 15 through-thickness holes in the PMMA interlayer, and positioned and held in place using the magnetic field focusing array. The through-thickness holes were then filled with 12.5% (w/v) PEGDMA precursor solution (MW = 1000, Polysciences, Inc.), which was then polymerized by exposing to 365 nm UV light at a power of 2.9 mW cm^–2^ (model XLE-1000 A/F, Spectroline) for 25–30 s.

In the second step, NIH/3T3 fibroblasts and GelMA precursor mixture were first mixed gently at the final concentration of 1.5 × 10^5 ^cells mL^–1^. 500 μL mixture was then pipetted onto the crosslinked PEGDMA within each of the 15 holes, and the PEGDMA was soaked in GelMA solution for 10 min. A glass cover slide, modified with 3-(trimethoxysilyl) propyl methacrylate (TMSPMA; Sigma-Aldrich) to conjugate with GelMA, was placed atop the molds to secure the fabricated μMASTs. Photopolymerization then proceeded using a custom-designed, commercially manufactured photomask (Shenzhen Qingyi Precision Mask Making Co., Ltd.). This photomask (Supplementary Fig. S16c) contained an array of 15 holes (0.8 mm diameter), and was placed atop the glass cover slide (Fig. S1 *D*) between the mold and the UV light source described above. The UV light source photo-crosslinked the GelMA layer for 15–25 s. 2-hydroxy-2-methylpropiophenone (TCI, Shanghai Development Co., Ltd.) was used as photoinitiator at the concentration of 0.05% (w/v) for crosslinking of both PEGDMA and GelMA. Both PEDMA and GelMA precursor solution were sterilized by filtration (0.22 μm pore size, Thermo Scientific Co., Ltd.) before using.

### Mechanical stretching of μMASTs

To design the magnetic field focusers and characterize the mechanical fields within μMASTs, we performed a series of numerical simulations using commercially available software (COMSOL Multiphysics 4.0a, Comsol Inc.). First, we predicted the magnetic field gradients experienced by the iron microspheres within the magnetically-actuated layers as a function of the separations between the ends of magnetic field focusers and the centers of iron microspheres. The magnetic force vector (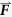) applied to each iron microsphere was estimated according to:


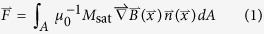


where *A* is the surface of the iron microsphere, 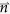 is the outward normal of *dA*, *μ*_0_ is the magnetic permeability of a vacuum, *M*_sat_ is the saturation moment of an individual iron microsphere, and 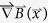 is the external magnetic field gradient tensor at position 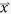.

This force was subsequently applied to a synthetic tissue as a uniform pressure on the upper surface; in-plane displacements were constrained to be zero on the upper surface, and all displacements were constrained to be zero on the lower surface. These boundary conditions are appropriate given the difference in stiffness between the PEGDMA and GelMA layers. GelMA were treated as an incompressible, isotropic Neo-Hookean with elastic modulus derived from experimental work described below.

### Experimental characterization of μMASTs

#### Mechanical characterization of μMASTs

To experimentally characterize magnetic force applied to iron microspheres in magnetically-actuated layers, the Stokes drag method was used. An iron microsphere was placed in a poly (ethylene oxide) solution of defined viscosity (PEO; Sigma-Aldrich), then subjected to a magnetic field. The speed of each iron microsphere was estimated as a function of separation between the end of the magnetic field focuser and the center of the iron microsphere from video taken through a 20× objective, acquired using a high-speed camera (Phantom Cinestream v.711, Vision Research, Co., Ltd.) at 3000 images per second. The exerted on the iron microsphere was then estimated using:


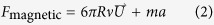


where the first term represents viscous drag, in which *v* is the dynamic viscosity of the PEO solution, *R* is the radius of the iron microsphere, and 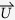 is the speed of the iron microsphere; and the second term represents an inertial force, in which m is the mass of the iron microsphere and *a* is the acceleration. A relationship between force and separation (Supplementary Fig. S2b) was calibrated from these experiments; as expected, the relationship was nearly inverse cubic. The dynamic viscosity v was estimated similarly from downward motions of iron microspheres release into a beaker of PEO solution in the absence of magnetic fields.

During stretching of μMASTs, microspheres were encapsulated in hydrogels and strain was estimated optically from images recorded using a high-resolution inverted fluorescence microscopy (IX-81, Olympus, Inc.) and analyzed using Image-Pro Plus (IPP; version 6.0, Media Cybernetics)^46^. Nominal strain, *ε*, was calculated in each layer as *ε* = *λ* − 1, *λ* is the ratio of the current to initial length of the layer. The average strain over the synthetic tissue layers was calculated by averaging the strain of 15 samples. The relationship between the Cauchy stress (force divided by current cross-sectional area) and this strain was found to be linear over a very broad range of strains for all GelMA concentrations tested. The elastic moduli of the synthetic tissues were derived from these relationships.

#### Structural characterization of μMASTs

For sample preparation, the hydrogel (control and strained) was firstly frozen at −80 °C for 24 h and then lyophilized by using a freeze-dryer (Heto PowerDry LL1500, Thermo Scientific) at room temperature for 12 h. The freeze-dried specimens were submerged into liquid nitrogen for about 5 minutes, and then fractured with a scalpel blade. The pore structure of hydrogels were sputter coated with platinum and then examined using a S-3000N, HITACHI scanning electron microscope (SEM). To test the porosity of hydrogels, hydrogel scaffolds were first thawed and hydrated for 24 hours. Hydrated scaffolds were weighed on a scale, and a Kimwipe was lightly applied to the scaffold surface for 40 s to wick away loosely held water, and the mass was again recorded. The interconnected volume was calculated as the mass of water wicked away divided by the total hydrated mass.

#### Cell viability and proliferation characterization

To measure cell viability and proliferation, cells encapsulated in synthetic tissues were stained with using a live/dead assay (Molecular Probes) following manufacturer’s instructions. Briefly, synthetic tissues were cut into several ~300 μm thickness circular cross-sectional slices using razor blades and incubated in a solution of 2 μg mL^–1^ calcein AM and 5 μg mL^–1^ propidium iodide at 37 °C for 20–30 min. Fluorescence microscopy was performed to identify cells that were living (stained green by calcein AM) and dead (stained red by propidium iodide). Cell nucleus were stained for cell number counting using IPP. Briefly, each florescent image of hydrogel slice was imported in IPP software. The “Area” option in “count/size-measure-select measurement” menu was then chosen. The cell number and was calculated through “count/size-count” option (Supplementary Fig. S17).

#### Cell spreading area characterization

To measure cell spreading area, F-actin stress fibers and the nuclei of cells were stained by fluorescein isothiocyanate (FITC) conjugated phalloidin (Acti-stain 488 phalloidin, Cytoskeleton, Inc.) and 4′, 6-diamidino-2-Phenylindole (DAPI; Invitrogen^TM^, Life Technologies, Inc.), respectively. Briefly, for stress fiber staining, cells in synthetic tissue slices were fixed by 4% formaldehyde (Sigma-Aldrich, St. Louis, MO, USA) for 15 min, permeabilized with 0.5% Triton X-100 (Sigma-Aldrich) for 5 min, and then incubated with 200 μL of 100 nM FITC phalloidin solution in the dark at room temperature for 30–40 min. For nuclear staining, cells were counterstained with 200 μL of 100 nM DAPI in DPBS for 30 s. The stained synthetic tissue slices were placed in a drop of antifade mounting medium (Invitrogen) on a glass slide and stored in the dark at 4 °C until imaging. Confocal fluorescence images were captured using a Zeiss LSM 700 microscope (Carl Zeiss). Cell spreading area was analyzed from these images using IPP.

#### Cell orientation analysis

The images acquired for cell spreading analysis were also analyzed to extract the orientation distribution and mean orientation of cells using the semi-automated “binarization-based extraction of alignment score” method described elsewhere^49^. Briefly, thresholded images were analyzed using a simple optimization scheme to identify the likelihood of cells possessing a dominant axis in directions ranging from 0 to 180°. The distributions were normalized.

#### MMP inhibition

To suppress the enzymatic activity of the matrix metalloproteinases (MMPs) secreted by NIH/3T3 fibroblasts, culture media was supplemented with a broad-spectrum MMP inhibitor BB94, (Selleck Chemicals, S7155) at a concentration of 400 μM, which has been previously shown to inhibit MMP expression and activation without significantly affecting cell function or viability^50^. The culture medium was exchanged per day. The samples were then stained for studying the effect of MMP inhibition on cell spreading and proliferation in μMASTs.

#### Cell apoptosis experiment

Cells in synthetic tissues were dissociated with 2.5% trypsin (Gibco BRL Life Technologies, Inc.), collected and suspended in DMEM. 5 μL annexin-V-FITC (Invitrogen^TM^, Life Technologies, Inc.) was then added into the cell suspension and incubated for 15 min at room temperature, followed by addition of 10 μL of propidium iodide (Invitrogen^TM^, Life Technologies, Inc.) and incubation for 5 min. Immediately after staining, a quantitative analysis was performed by using a flow cytometer (Becton, Dickinson and Company). Data were analyzed with FlowJo software (Tomy Digital Biology).

## Additional Information

**How to cite this article**: Li, Y. *et al*. An approach to quantifying 3D responses of cells to extreme strain. *Sci. Rep.* **6**, 19550; doi: 10.1038/srep19550 (2016).

## Supplementary Material

Supplementary Information

## References

[b1] Sherwood L. & Pysiology H. From Cells to Systems. (Human Physiology, 2010).

[b2] Parameswaran H. . Three-dimensional measurement of alveolar airspace volumes in normal and emphysematous lungs using micro-CT. J Appl Physiol 107, 583–592 (2009). 19541732 10.1152/japplphysiol.91227.2008 PMC2724324

[b3] Ahmed J., Mallick I. H. & Ahmad S. M. Rupture of urinary bladder: a case report and review of literature. Cases J. 2, 7004 (2009). 19829892 10.1186/1757-1626-2-7004 PMC2740071

[b4] Huffman J. L., Schraut W. & Bagley D. H. Atraumatic Perforation of Bladder - Necessary Differential in Evaluation of Acute Condition of Abdomen. Urology 22, 30–35 (1983). 6868247 10.1016/0090-4295(83)90341-2

[b5] Shumaker B. P., Pontes J. E. & Pierce J. M. Idiopathic Rupture of Bladder. Urology 15, 566–568 (1980). 7394981 10.1016/0090-4295(80)90367-2

[b6] Elson E. L. & Genin G. M. The role of mechanics in actin stress fiber kinetics. Exp. Cell. Res. 319, 2490–2500 (2013). 23906923 10.1016/j.yexcr.2013.06.017 PMC3955124

[b7] Legant W. R. . Measurement of mechanical tractions exerted by cells in three-dimensional matrices. Nat. Methods. 7, 969–971 (2010). 21076420 10.1038/nmeth.1531 PMC3056435

[b8] Ross T. D. . Integrins in mechanotransduction. Curr. Opin. Cell. Biol. 25, 613–618 (2013). 23797029 10.1016/j.ceb.2013.05.006 PMC3757118

[b9] Trepat X. . Universal physical responses to stretch in the living cell. Nature 447, 592–595 (2007). 17538621 10.1038/nature05824 PMC2440511

[b10] Lee S. L. . Physically-Induced Cytoskeleton Remodeling of Cells in Three-Dimensional Culture. Plos One 7, e45512 (2012). 23300512 10.1371/journal.pone.0045512 PMC3531413

[b11] Elson E. L. Cellular Mechanics as an Indicator of Cytoskeletal Structure and Function. Annu. Rev. Biophys. Bio. 17, 397–430 (1988). 10.1146/annurev.bb.17.060188.002145 3293593

[b12] Eschenhagen T. . Three-dimensional reconstitution of embryonic cardiomyocytes in a collagen matrix: a new heart muscle model system. Faseb. J. 11, 683–694 (1997). 9240969 10.1096/fasebj.11.8.9240969

[b13] Gossett D. R. . Hydrodynamic stretching of single cells for large population mechanical phenotyping. Proc. Natl. Acad. Sci. USA 109, 7630–7635 (2012). 22547795 10.1073/pnas.1200107109 PMC3356639

[b14] Haga J. H., Li Y. S. J. & Chien S. Molecular basis of the effects of mechanical stretch on vascular smooth muscle cells. J. Biomech. 41, 2331–2331 (2008). 10.1016/j.jbiomech.2006.04.011 16867303

[b15] Yang C., Tibbitt M. W., Basta L. & Anseth K. S. Mechanical memory and dosing influence stem cell fate. Nat. Mater. 13, 645–652 (2014). 24633344 10.1038/nmat3889 PMC4031270

[b16] Tan J. L. . Cells lying on a bed of microneedles: An approach to isolate mechanical force. Proc. Natl. Acad. Sci. USA 100, 1484–1489 (2003). 12552122 10.1073/pnas.0235407100 PMC149857

[b17] Trepat X. . Physical forces during collective cell migration. Nat. Phys. 5, 426–430 (2009).

[b18] Kaunas R., Usami S. & Chien S. Regulation of stretch-induced JNK activation by stress fiber orientation. Cell Signal 18, 1924–1931 (2006). 16581230 10.1016/j.cellsig.2006.02.008

[b19] Cukierman E., Pankov R., Stevens D. R. & Yamada K. M. Taking cell-matrix adhesions to the third dimension. Science 294, 1708–1712 (2001). 11721053 10.1126/science.1064829

[b20] Ghibaudo M., Di Meglio J. M., Hersen P. & Ladoux B. Mechanics of cell spreading within 3D-micropatterned environments. Lab Chip 11, 805–812 (2011). 21132213 10.1039/c0lc00221f

[b21] Lee J., Abdeen A. A., Zhang D. & Kilian K. A. Directing stem cell fate on hydrogel substrates by controlling cell geometry, matrix mechanics and adhesion ligand composition. Biomaterials 34, 8140–8148 (2013). 23932245 10.1016/j.biomaterials.2013.07.074

[b22] Screen H. R. C., Lee D. A., Bader D. L. & Shelton J. C. An investigation into the effects of the hierarchical structure of tendon fascicles on micromechanical properties. P. I. Mech. Eng. H 218, 109–119 (2004). 10.1243/095441104322984004 15116898

[b23] Marquez J. P., Elson E. L. & Genin G. M. Whole cell mechanics of contractile fibroblasts: relations between effective cellular and extracellular matrix moduli. Philos. T. R. Soc. A 368, 635–654 (2010). 10.1098/rsta.2009.0240 PMC3263794 20047943

[b24] Marquez J. P., Genin G. M., Zahalak G. I. & Elson E. L. The relationship between cell and tissue strain in three-dimensional bio-artificial tissues. Biophys. J. 88, 778–789 (2005). 15596491 10.1529/biophysj.104.041947 PMC1305155

[b25] Wakatsuki T., Kolodney M. S., Zahalak G. I. & Elson E. L. Cell mechanics studied by a reconstituted model tissue. Biophys. J. 79, 2353–2368 (2000). 11053115 10.1016/S0006-3495(00)76481-2 PMC1301123

[b26] Eschenhagen T. & Zimmermann W. H. Engineering myocardial tissue. Circ. Res. 97, 1220–1231 (2005). 16339494 10.1161/01.RES.0000196562.73231.7d

[b27] Li Y. . Engineering cell alignment *in vitro*. Biotechnol. Adv. 32, 347–365 (2014). 24269848 10.1016/j.biotechadv.2013.11.007

[b28] Li Y. . Chinese-Noodle-Inspired Muscle Myofiber Fabrication. Adv. Funct. Mater. doi: 10.1002/adfm.201502018 (2015).

[b29] Wang L. . Hydrogel-based methods for engineering cellular microenvironment with spatiotemporal gradients. Crit. Rev. Biotechnol. 1, 1–13 (2015). 10.3109/07388551.2014.993588 25641330

[b30] Legant W. R. . Microfabricated tissue gauges to measure and manipulate forces from 3D microtissues. Proc. Natl. Acad. Sci. USA 106, 10097–10102 (2009). 19541627 10.1073/pnas.0900174106 PMC2700905

[b31] Vandenburgh H. . Drug-screening platform based on the contractility of tissue-engineered muscle. Muscle Nerve 37, 438–447 (2008). 18236465 10.1002/mus.20931

[b32] Zhao R. G., Boudou T., Wang W. G., Chen C. S. & Reich D. H. Decoupling Cell and Matrix Mechanics in Engineered Microtissues Using Magnetically Actuated Microcantilevers. Adv. Mater. 25, 1699–1705 (2013). 23355085 10.1002/adma.201203585 PMC4037409

[b33] Foolen J., Deshpande V. S., Kanters F. M. W. & Baaijens F. P. T. The influence of matrix integrity on stress-fiber remodeling in 3D. Biomaterials 33, 7508–7518 (2012). 22818650 10.1016/j.biomaterials.2012.06.103

[b34] Silva R., Fabry B. & Boccaccini A. R. Fibrous protein-based hydrogels for cell encapsulation. Biomaterials 35, 6727–6738 (2014). 24836951 10.1016/j.biomaterials.2014.04.078

[b35] Brown A. E. X., Litvinov R. I., Discher D. E., Purohit P. K. & Weisel J. W. Multiscale Mechanics of Fibrin Polymer: Gel Stretching with Protein Unfolding and Loss of Water. Science 325, 741–744 (2009). 19661428 10.1126/science.1172484 PMC2846107

[b36] Da Cunha C. B. . Influence of the stiffness of three-dimensional alginate/collagen-I interpenetrating networks on fibroblast biology. Biomaterials 35, 8927–8936 (2014). 25047628 10.1016/j.biomaterials.2014.06.047

[b37] Huebsch N. . Harnessing traction-mediated manipulation of the cell/matrix interface to control stem-cell fate. Nat. Mater. 9, 518–526 (2010). 20418863 10.1038/nmat2732 PMC2919753

[b38] Galie P., Russell M., Westfall M. & Stegemann J. Interstitial fluid flow and cyclic strain differentially regulate cardiac fibroblast activation via AT1R and TGF-β1. Exp. Cell. Res. 318, 75–84 (2012). 22020089 10.1016/j.yexcr.2011.10.008 PMC3221916

[b39] Nakayama A. . High mechanical strength double-network hydrogel with bacterial cellulose. Adv. Funct. Mater. 14, 1124–1128 (2004).

[b40] Solon J., Levental I., Sengupta K., Georges P. C. & Janmey P. A. Fibroblast adaptation and stiffness matching to soft elastic substrates. *Biophys. J*. 93, 4453–4461 (2007). 18045965 10.1529/biophysj.106.101386 PMC2098710

[b41] Egeblad M. & Werb Z. New functions for the matrix metalloproteinases in cancer progression. Nat. Rev. Cancer 2, 161–174 (2002). 11990853 10.1038/nrc745

[b42] Hinz B., Mastrangelo D., Iselin C. E., Chaponnier C. & Gabbiani G. Mechanical tension controls granulation tissue contractile activity and myofibroblast differentiation. Am. J. Pathol. 159, 1009–1020 (2001). 11549593 10.1016/S0002-9440(10)61776-2 PMC1850455

[b43] Zemel A., Bischofs I. B. & Safran S. A. Active elasticity of gels with contractile cells. Phys. Rev. Lett. 97, 123–128 (2006). 10.1103/PhysRevLett.97.128103 17026002

[b44] Greiner A. M., Chen H., Spatz J. P. & Kemkemer R. Cyclic Tensile Strain Controls Cell Shape and Directs Actin Stress Fiber Formation and Focal Adhesion Alignment in Spreading Cells. Plos One 8, e77328 (2013). 24204809 10.1371/journal.pone.0077328 PMC3810461

[b45] Balaban N. Q. . Force and focal adhesion assembly: a close relationship studied using elastic micropatterned substrates. Nat. Cell. Biol. 3, 466–472 (2001). 11331874 10.1038/35074532

[b46] Wang L. . Patterning Cellular Alignment through Stretching Hydrogels with Programmable Strain Gradients. Acs Appl. Mater. Inter. 7, 15088–15097 (2015). 10.1021/acsami.5b04450 26079936

[b47] Nichol J. W. . Cell-laden microengineered gelatin methacrylate hydrogels. Biomaterials 31, 5536–5544 (2010). 20417964 10.1016/j.biomaterials.2010.03.064 PMC2878615

[b48] Marquez J. P., Genin G. M. & Elson E. L. On the application of strain factors for approximation of the contribution of anisotropic cells to the mechanics of a tissue construct. J. Biomech. 39, 2145–2151 (2006). 16055135 10.1016/j.jbiomech.2005.06.010

[b49] Feng X., Beyazoglu T., Hefner E., Gurkan U. A. & Demirci U. Automated and Adaptable Quantification of Cellular Alignment from Microscopic Images for Tissue Engineering Applications. Tissue Eng. Part C-Me. 17, 641–649 (2011). 10.1089/ten.tec.2011.0038 PMC3103056 21370940

[b50] Aubin H. . Directed 3D cell alignment and elongation in microengineered hydrogels. Biomaterials 31, 6941–6951 (2010). 20638973 10.1016/j.biomaterials.2010.05.056 PMC2908986

