# Supplementary material for: An approach to quantifying 3D responses of cells to extreme strain
Source: Sci Rep. 2016 Feb 18;6:19550. doi: 10.1038/srep19550 (PMC4757889; doi:10.1038/srep19550)
Supplement: Supplementary Information [file srep19550-s1.doc]

**An approach to quantifying 3D responses of cells to extreme strain**

Yuhui Li^1,2#^, Guoyou Huang^1,2#^, Moxiao Li^2^, Lin Wang^1,2,3^, Elliot L. Elson^2,3,5^, Tian Jian Lu^2^, Guy M. Genin^2,4,5*^, Feng Xu^1,2*^

*^1^ The Key Laboratory of Biomedical Information Engineering of Ministry of Education, School of Life Science and Technology, Xi’an Jiaotong University, Xi’an 710049, China*

*^2^ Bioinspired Engineering and Biomechanics Center, Xi’an Jiaotong University, Xi’an 710049, China*

*^3^ Department of Biochemistry and Molecular Biophysics, and ^4^ Department of Neurological Surgery, Washington University School of Medicine, Saint Louis, Missouri 63110, USA*

*^5^ Department of Mechanical Engineering and Materials Science, Washington University, Saint Louis, Missouri 63130, USA*

*^#^ Y.L. and G.H. contributed equally to this work*

*^*^ Correspondence should be addressed to* [*fengxu@mail.xjtu.edu.cn*](mailto:fengxu@mail.xjtu.edu.cn)*, genin@wustl.edu*

**Supplementary Information**

| **Supplementary Figure 1** | Fabrication of μMASTs. |
| --- | --- |
| **Supplementary Figure 2** | Characterization of the mechanical effects of μMAST fabrication protocols. |
| **Supplementary Figure 3** | Swelling ratios of the μMASTs. |
| **Supplementary Figure 4** | Specific magnetization of a single iron microsphere. |
| **Supplementary Figure 5** | Magnetic field focusing system. |
| **Supplementary Figure 6** | Magnetic fields did not affect the observed cellular responses. |
| **Supplementary Figure 7** | Elastic modulus of μMASTs (6kPa group) at day 1, 3 and 5 of culturing, respectively. |
| **Supplementary Figure 8** | Elastic modulus of μMASTs. |
| **Supplementary Figure 9** | Effect of MMP inhibition on cell spreading and proliferation in μMASTs. |
| **Supplementary Figure 10** | Polarization of cells in strained μMASTs with different modulus. |
| **Supplementary Figure 11** | Effect of MMP inhibition on cell polarization in μMASTs. |
| **Supplementary Figure 12** | Proliferation of cells in strained μMASTs. |
| **Supplementary Figure 13** | Immunofluorescence staining and fluorescence analysis of apoptotic cells in μMASTs after straining. |
| **Supplementary Figure 14** | Structural characterization of μMASTs. |
| **Supplementary Figure 15** | Quantification of nuclear morphology. |
| **Supplementary Figure 16** | PMMA mold and photomask. |
| **Supplementary Figure 17** | Quantification methods of cell numbers in per μMAST. |


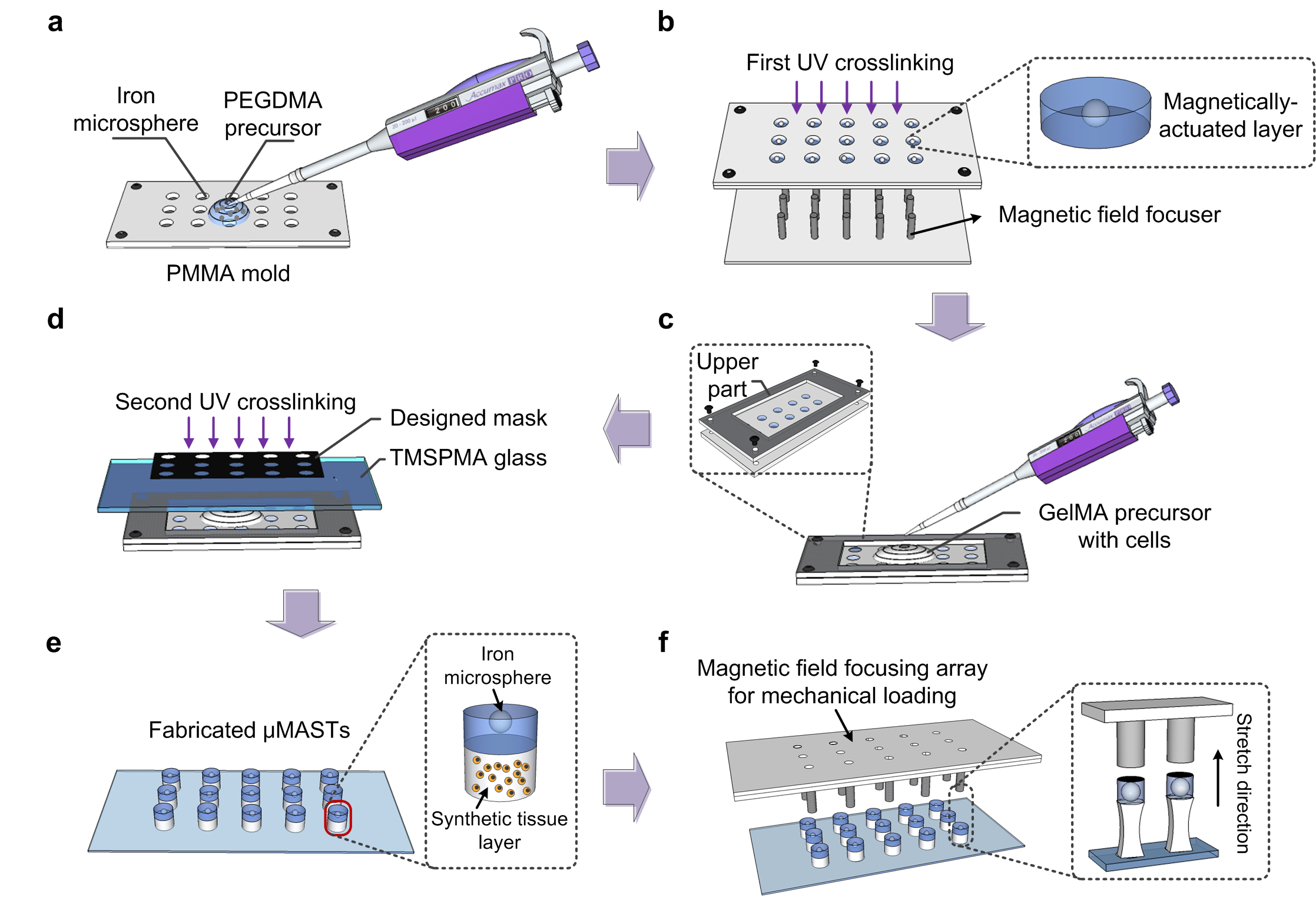


**Supplementary Figure 1 |** Fabrication of μMASTs. (**a**) PEGDMA precursor was poured into a custom PMMA mold, filling the 15 through-thickness holes in the “interlayer” (cf. **Supplementary Figure 12**). An iron microsphere was positioned in the center of these holes using the magnetic field focusing array. (**b**) PEGDMA was cross-linked using UV light to form magnetically-actuated layers while the iron microspheres continued to be held in place by the magnetic field focusing array. (**c**) The PMMA “cover” (cf. **Supplementary Figure 12**) was affixed atop the interlayer, and the rectangular cut-out in its center was filled with a solution consisting of GelMA precursor and cells. (**d**) The GelMA precursor solution within the cover was then sealed with a TMSPMA-modified glass slide. The glass slide was covered with a photomask. The assembly was then exposed to UV light through the photomask to crosslink the GelMA precursor solution. (**e**) μMASTs were formed, each composed of magnetically-actuated layer and synthetic tissue layer. (**f**) The magnetic field focusing array was applied to stretch μMASTs in experiments designed to define cellular mechanosensitivity in 3D culture. (The schematic was drawn by Yuhui Li using SketchUp Pro 8.0)

**
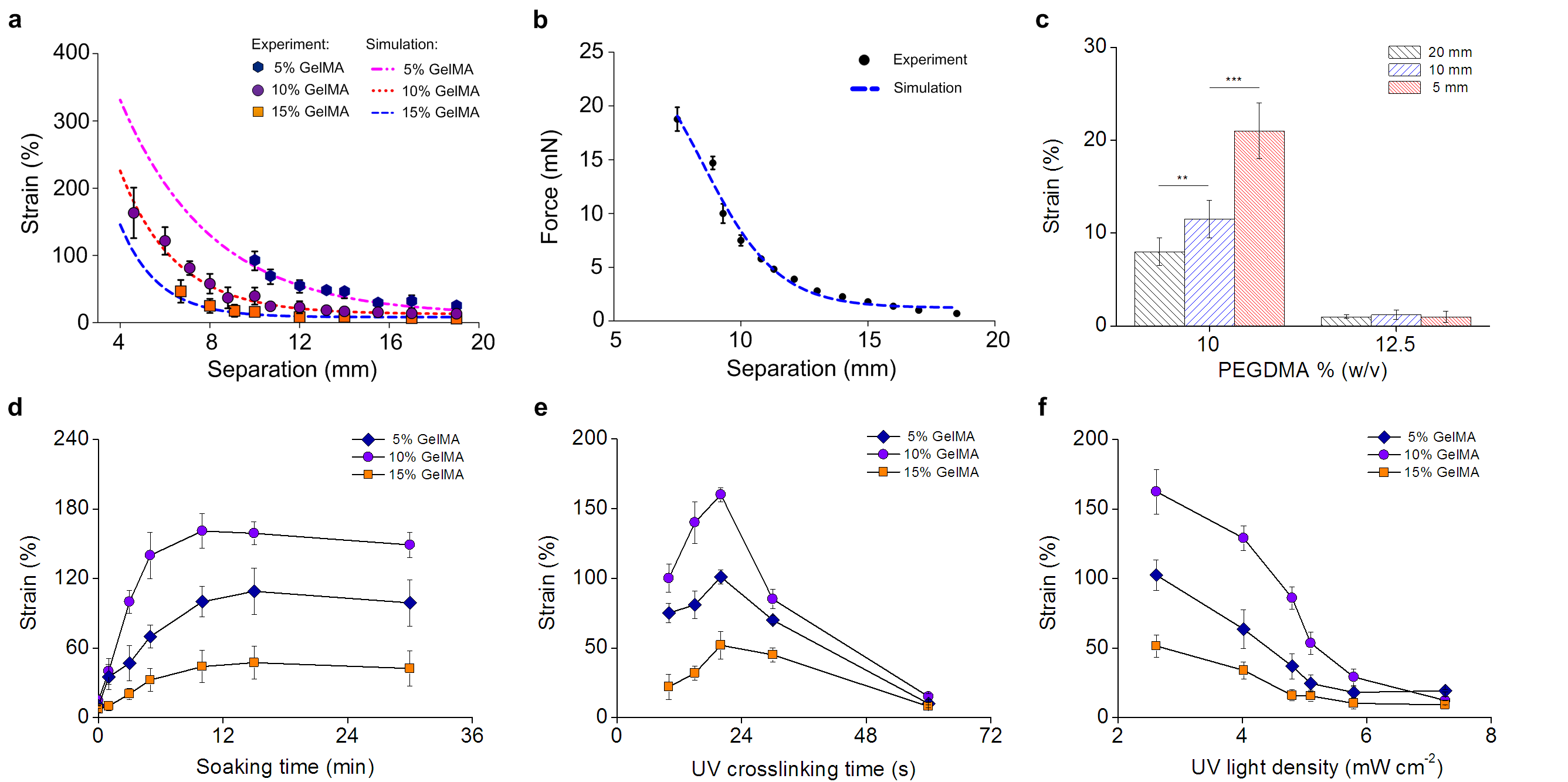
**

**Supplementary Figure 2** | Characterization of the mechanical effects of μMAST fabrication protocols. (**a**) The degree of straining of the synthetic tissue layers decreased with increasing separation between the magnetic field focuser and the iron microspheres (symbols: experiment; lines: simulation). (**b**) The magnetic force was an approximately inverse-cubic function of this separation (symbols: experiment; lines: simulation). (**c**) We minimized deformation of the magnetically-actuated layer by modifying the PEGDMA concentration. Above 12.5% (w/v) PEGDMA, this deformation was negligible relative to that of the synthetic tissue layer. Shown are nominal strains in the magnetically-actuated layer over the range of mechanical testing parameters used (separation distances between the ends of the magnetic field focusers and the centers of iron microspheres ranging from 5 to 20 mm). Error bars, s.d. (**a**: 5 ≤ n ≤ 10 for each data point; **b**: 10 ≤ n ≤ 12 for each data point; **c** 5 ≤ n ≤ 10 for each concentration of PEGDMA or data point, ***p* < 0.01, ****p* < 0.001).


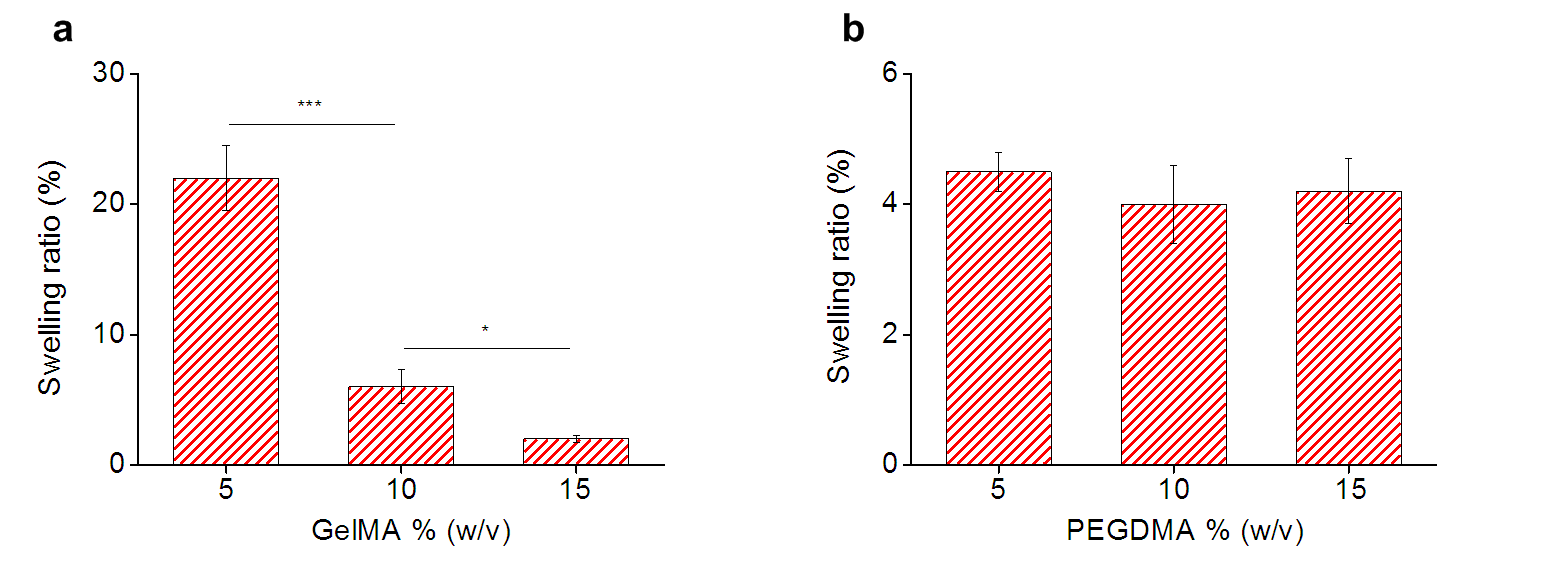


**Supplementary Figure 3** | Swelling ratios of μMASTs. μMASTs swelled when immersed in cell culture medium. To minimize the mechanical consequences of this, the GelMA precursor solution concentration was varied to match the swelling of the PEGDMA. (**a**) Swelling ratio of synthetic tissue layer as a function of GelMA concentration. (**b**) Swelling ratio of magnetically-actuated layer as a function of PEGDMA concentration. Error bars, s.d. (10 ≤ n ≤ 15 for each concentration of GelMA or PEGDMA, **p* < 0.05, ****p* < 0.001).


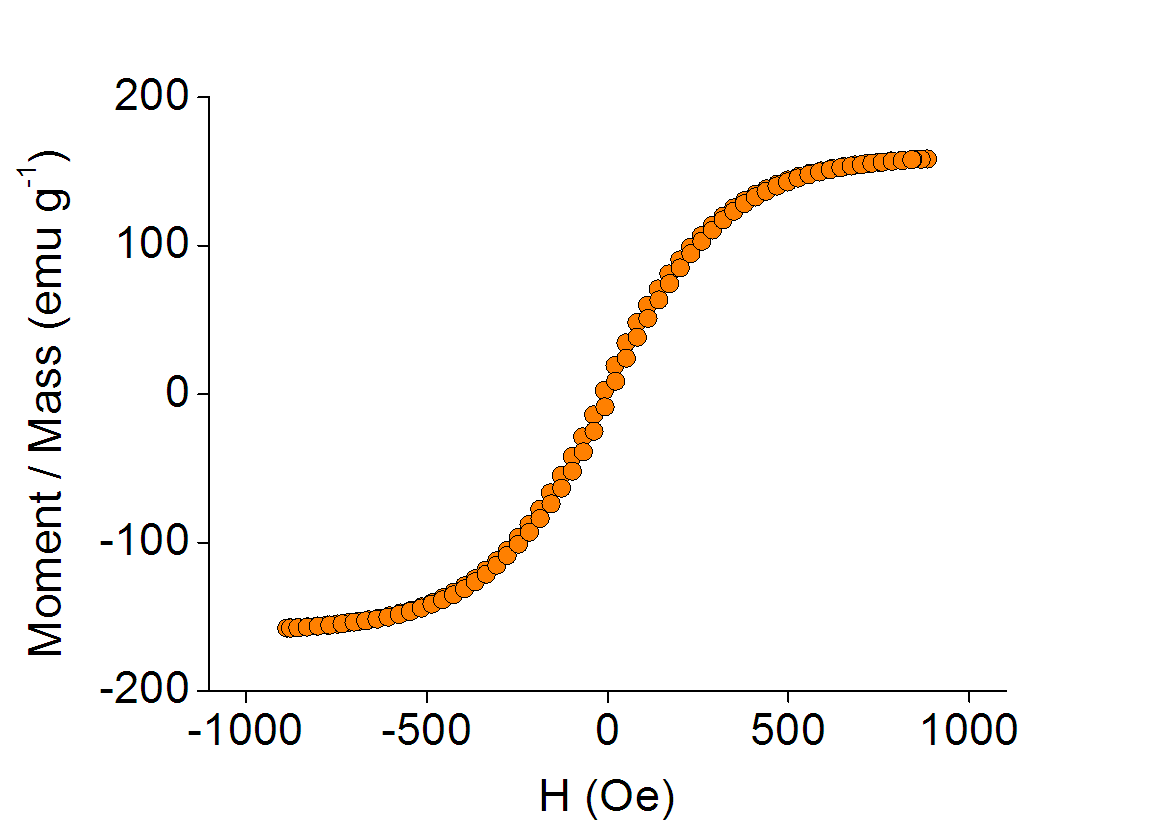


**Supplementary Figure 4** | Specific magnetization of a single iron microsphere. Magnetic moment per unitmass showed very little hysteresis. From these measurements, the saturation moment was estimated as *M_sat_* = 156 emu g^-1^.

**
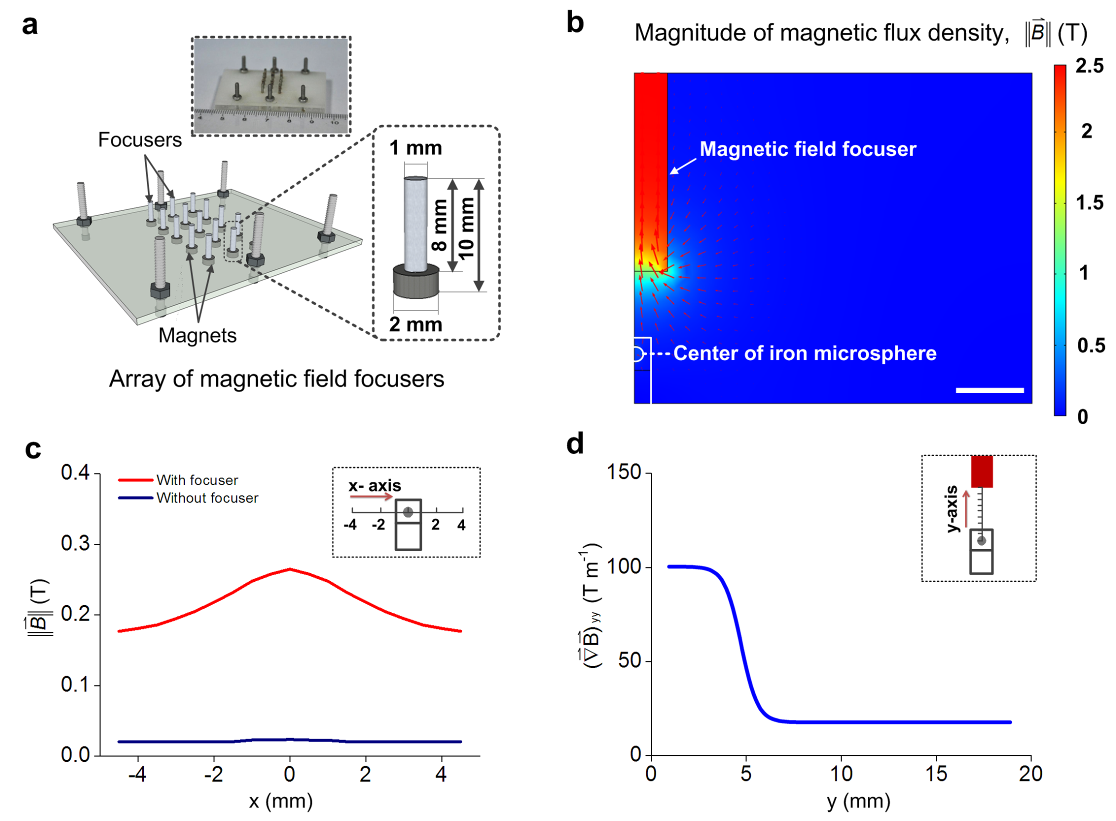
**

**Supplementary Figure 5** | Magnetic field focusing system. (**a**) Arrays of magnetic field focusing devices were constructed, each consisting of a permanent magnet and an iron focusing wire. (**b** and **c**) Simulations showed that the focusing wire concentrated and amplified magnetic flux density near its free end. Shown are a contour plot and graph of the magnitude of the magnetic flux density. (**d**) These simulations also provided a calibration curve that was used in conjunction with calibration measurements to control deformation of μMASTs. The gradient in the magnetic flux density is a tensor; the yy-component of that tensor along the centerline (x = z = 0) is plotted. Scale bar: 1 mm. (The schematic was drawn by Yuhui Li using SketchUp Pro 8.0)


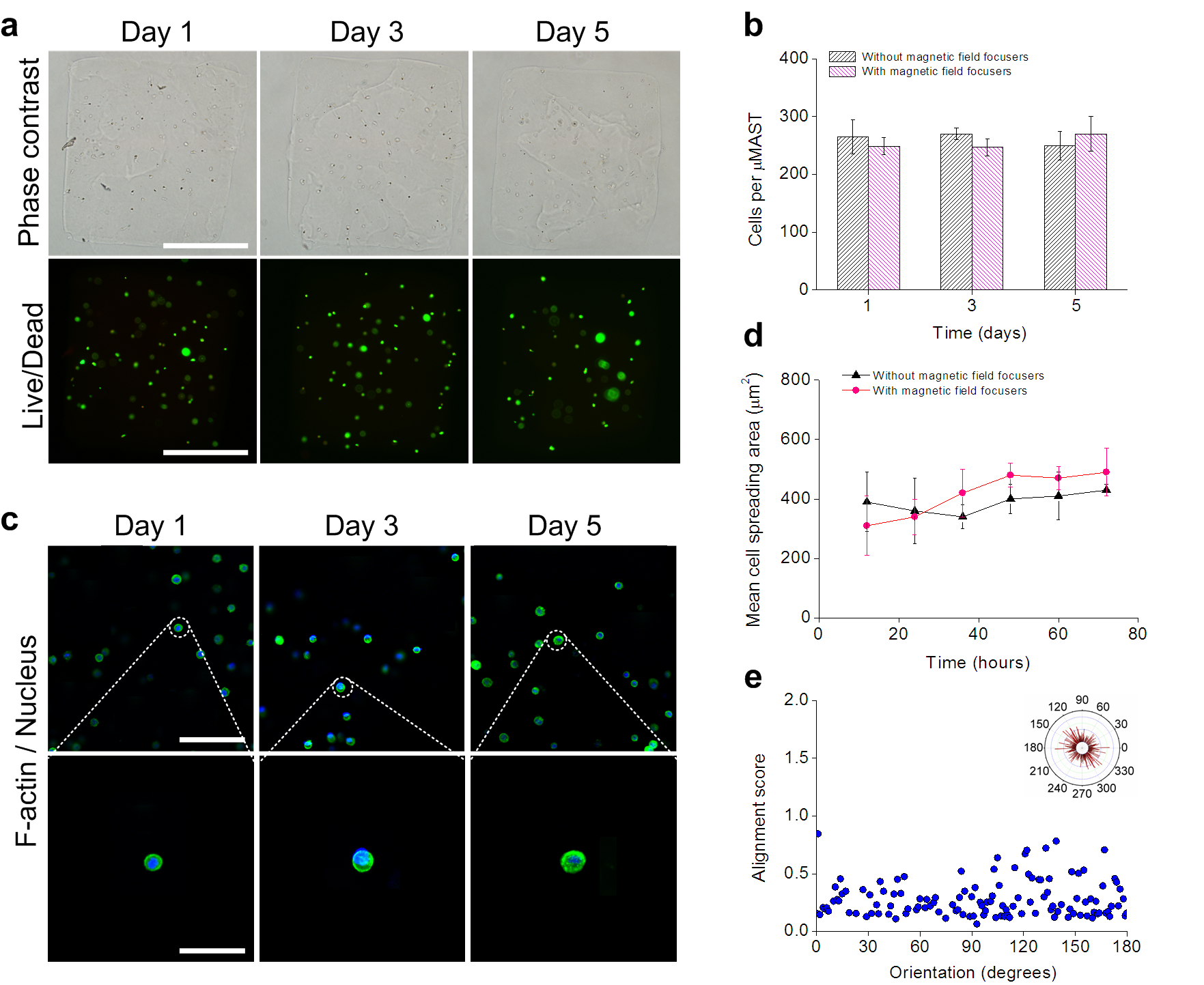


**Supplementary Figure 6** | Magnetic fields did not affect the observed cellular responses. To rule out magnetic fields as a cause of the observed cellular responses, control μMASTs were constructed without iron microspheres and then placed beneath the array of magnetic field focusers at the maximum strength used in experiments for defined time intervals. No effects of magnetic fields were observed relative to additional control μMASTs that were not placed in magnetic fields: (**a**) Phase contrast (upper) and live/dead fluorescence (bottom) images showed no discernable or measurable changes over 1, 3, or 5 days of culture in the focused magnetic field. (**b**) No effect of magnetic fields on proliferation was observed. (**c**) Confocal fluorescence images showed no discernable morphological responses to magnetic fields after 1, 3, or 5 days of culture (green: F-actin (phalloidin); blue: nuclei (DAPI)). (**d**) No statistically significant effect on cell spreading area was observed. (**e**) No preferred direction of cell polarization emerged in unstrained μMASTs subjected to a magnetic field for 5 days. Synthetic tissue modulus: 6 kPa. Error bars, s.d. (5 ≤ n ≤ 10 μMASTs for each bar or data point). Scale bars: (**a**) 500 μm, (**b**) upper panels: 100 μm; lower panels: 50 μm.


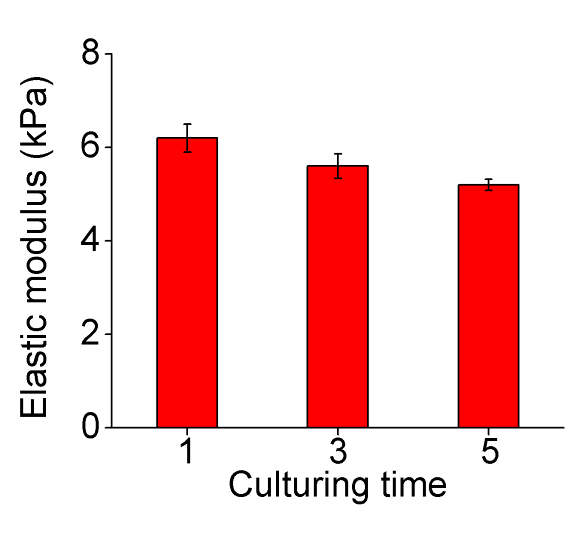


**Supplementary Figure 7** | Elastic modulus of μMASTs (6kPa group) at day 1, 3 and 5 of culturing, respectively. n=10 μMASTs.

**
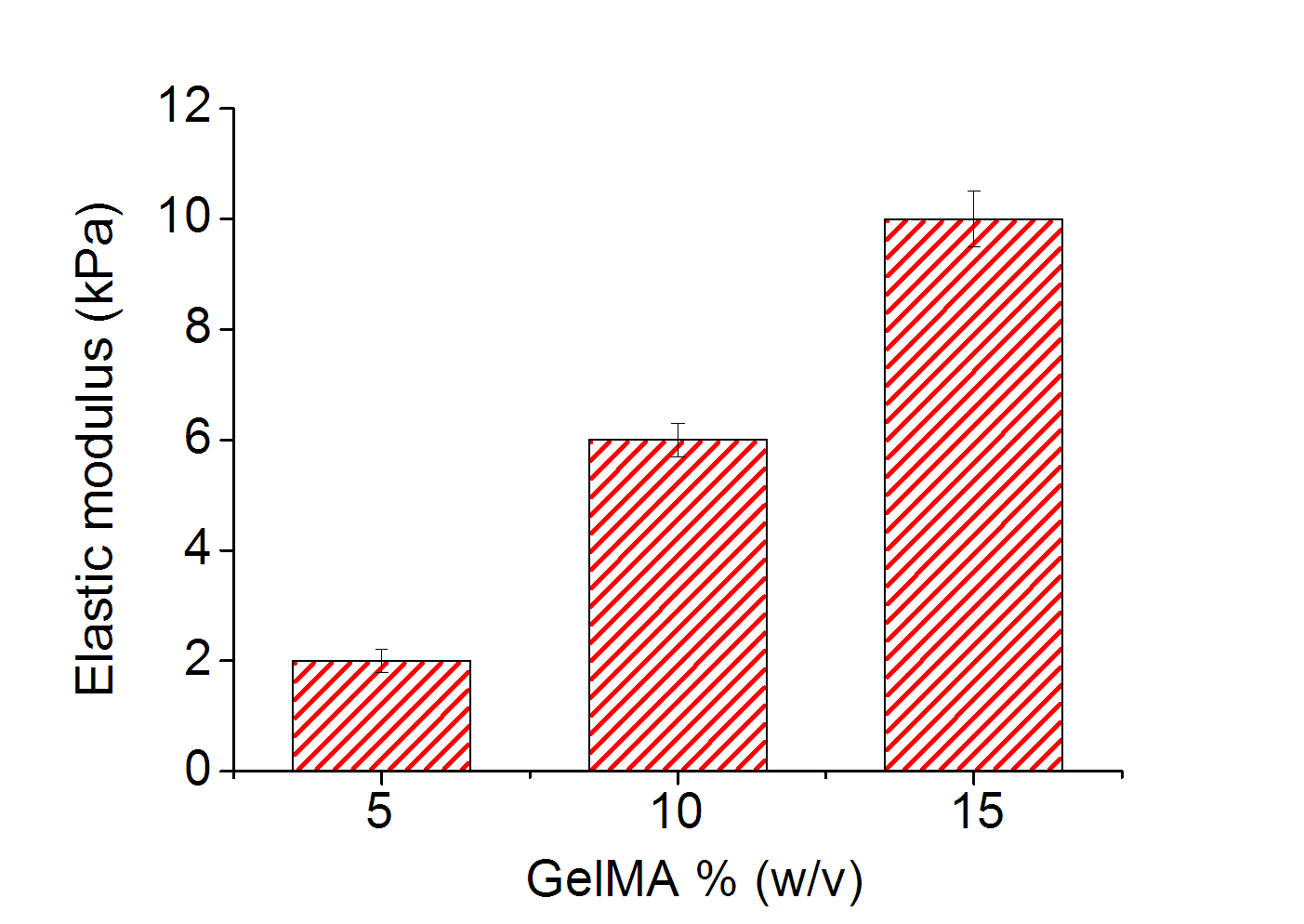
**

**Supplementary Figure 8** | Elastic modulus of μMASTs. Elastic modulus of the synthetic tissue layers could be controlled by modulating the GelMA precursor concentration. Error bars, s.d. (10 ≤ n ≤ 15 for each concentration of GelMA).


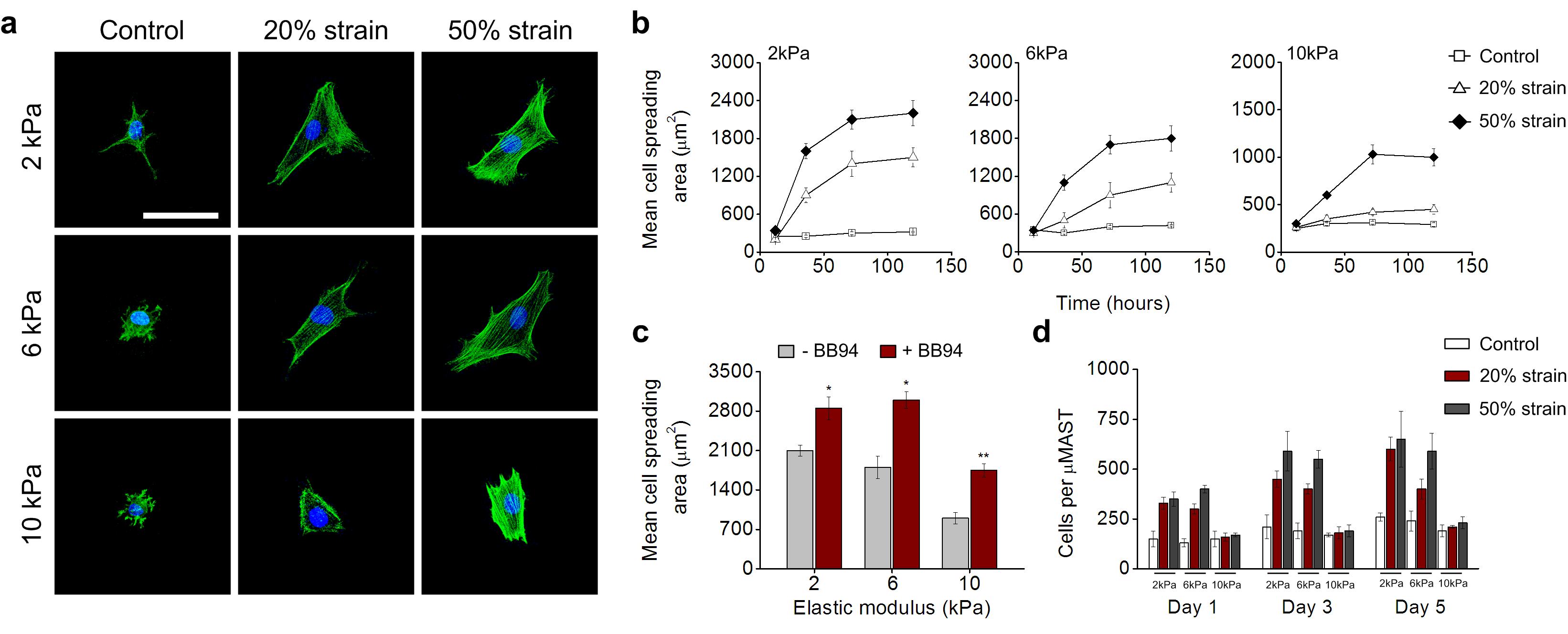


**Supplementary Figure 9** | Effect of MMP inhibition on cell spreading and proliferation in μMASTs. (**a**) Confocal fluorescence images of cells in μMASTs with BB94 inhibitor after 3 days of straining to different levels (Green: F-actin (phalloidin); blue: nuclei (DAPI)). (**b**) Mean cell spreading area showed the consistent increased trend with increasing strain levels compared to groups without BB94 inhibitor. (**c-d**) The maximum mean cell spreading area in three kinds of μMASTs with 50% strain is 1850 ± 200 μm^2^ (**c**) and the cell numbers per μMAST is 620 ± 120 after 5 days of culturing (**d**), which is lower than group without BB94 (mean cell spreading area is 2550 ± 100 μm^2^ and cell numbers per μMAST is 750 ± 50). Error bars, s.d. (n=10 μMASTs for each strain level, **p* < 0.05, ****p* < 0.001). Scale bars: 50 μm.

**
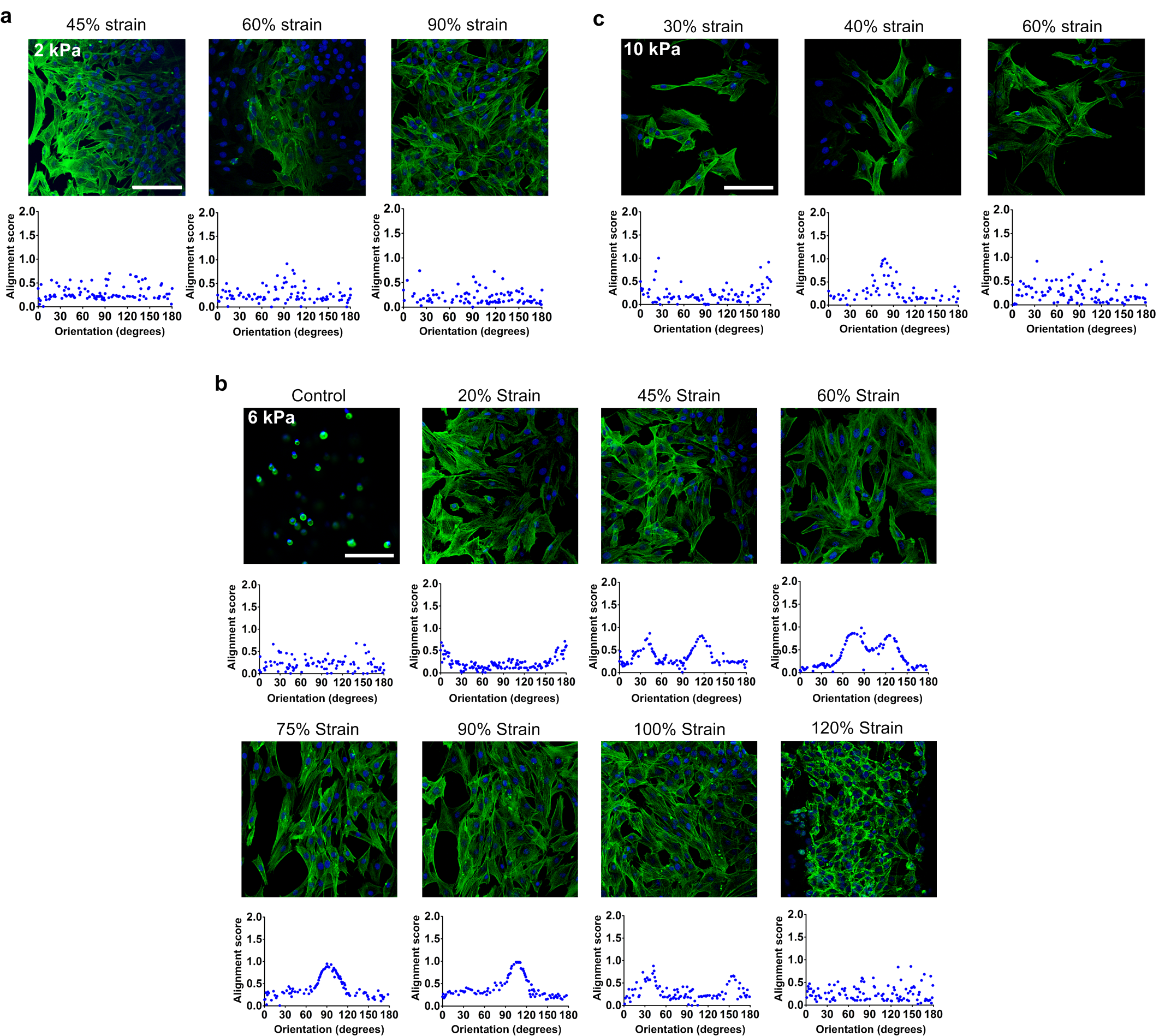
**

**Supplementary Figure 10** | Polarization of cells in strained μMASTs with different modulus. (**a**) Confocal fluorescence images of cells in 2 kPa synthetic tissues after 5 days of straining to prescribed levels (green: F-actin/phalloidin; blue: nuclei/DAPI). Cells oriented parallel to the applied stretch at 60% strain levels. (**b**) Confocal fluorescence images of cells in 6 kPa μMASTs after 5 days of straining to different levels. The control group represented unstrained μMASTs. Cells oriented perpendicular to the applied stretch at lower strain levels and parallel to the applied stretch at higher strain levels (75% and 90%). At the lowest and highest levels of stretch, no dominant polarization was observed. (**c**) Confocal fluorescence images of cells in 10 kPa synthetic tissues after 5 days of straining to prescribed levels. Cells oriented parallel to the applied stretch at 40% strain levels. 10 ≤ n ≤ 15 μMASTs for each strain level. Scale bar: 100 μm.

*
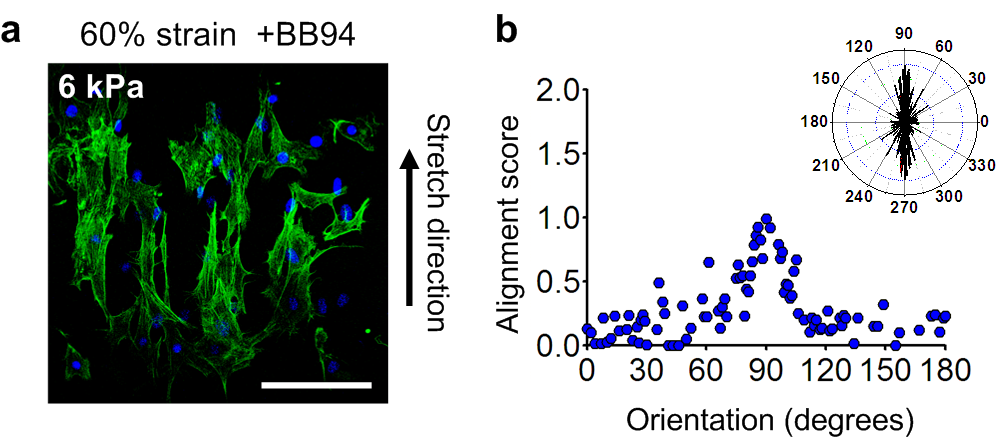
*

**Supplementary Figure 11** | Effect of MMP inhibition on cell polarization in μMASTs. (**a**) Confocal fluorescence images of cells in 6 kPa synthetic tissues with MMP inhibitor after 5 days of straining to prescribed levels (green: F-actin/phalloidin; blue: nuclei/DAPI). Cells reoriented parallel to the applied stretch at 60% strain levels. (**b**) Cell alignment quantification results. n=10 μMASTs. Scale bar: 100 μm.

**
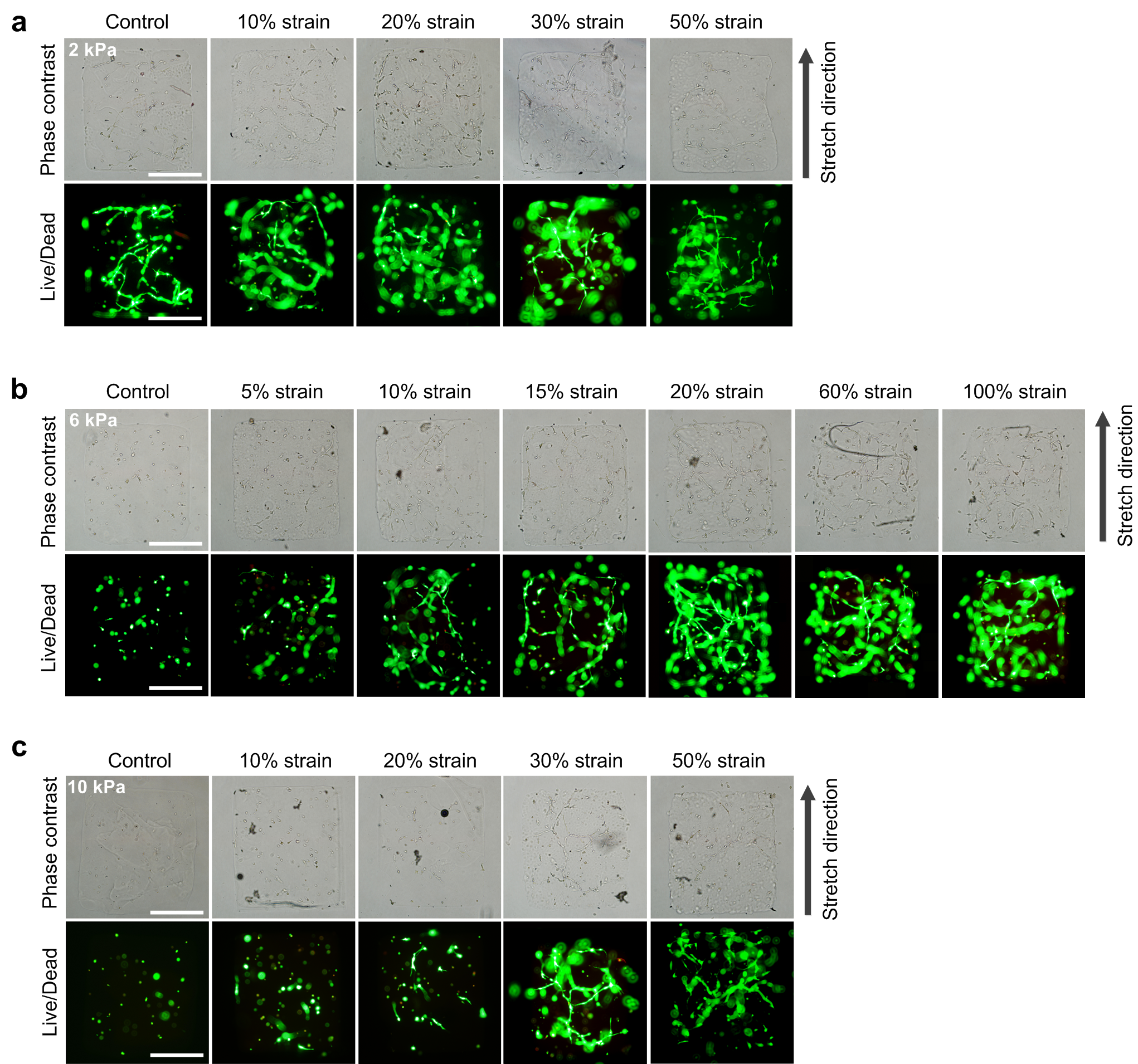
**

**Supplementary Figure 12** | Proliferation of cells in strained μMASTs. (**a**) Phase contrast (upper) and live/dead fluorescence (bottom) images of cells in 2 kPa μMASTs after 3 days of straining to different levels. Live/dead fluorescence images showed overwhelmingly live (green) cells and few dead (red) cells. (**b** and **c**) Phase contrast (upper) and live/dead fluorescence (bottom) images of cells in (**b**) 6 kPa and (**c**) 10 kPa μMASTs after 3 days of straining to different levels. Scale bars: 500 μm.


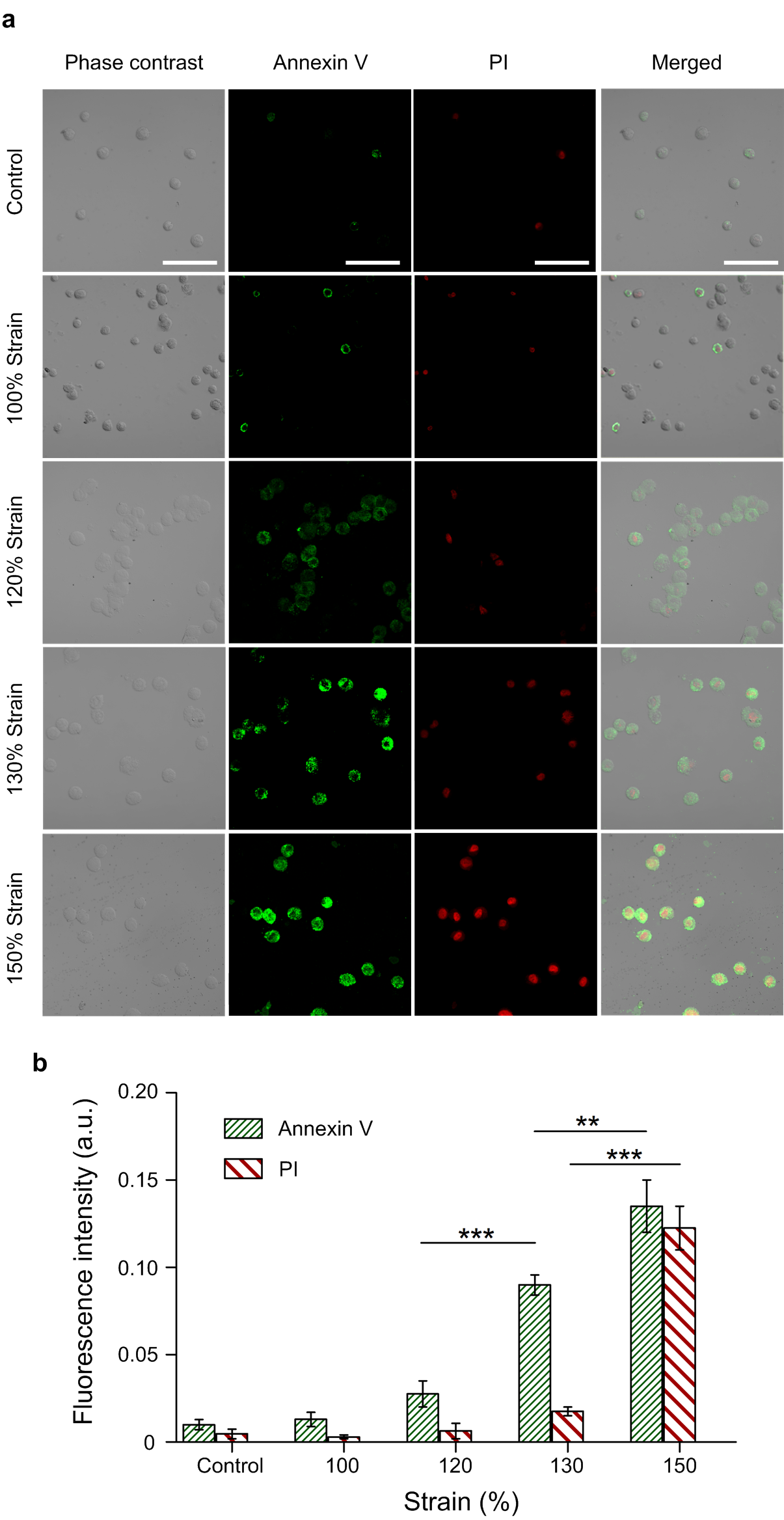


**Supplementary Figure 13** | Immunofluorescence staining and fluorescence analysis of apoptotic cells in μMASTs after straining. (**a**) Annexin V and PI staining after 5 days of straining. (**b**) Fluorescence intensity profiles of Annexin V and PI in cells after straining. Synthetic tissue modulus: 6 kPa. Error bars, s.d. (10 ≤ n ≤ 15 μMASTs for each strain level, ***p* < 0.01, ****p* < 0.001). Scale bars: 100 μm.

*
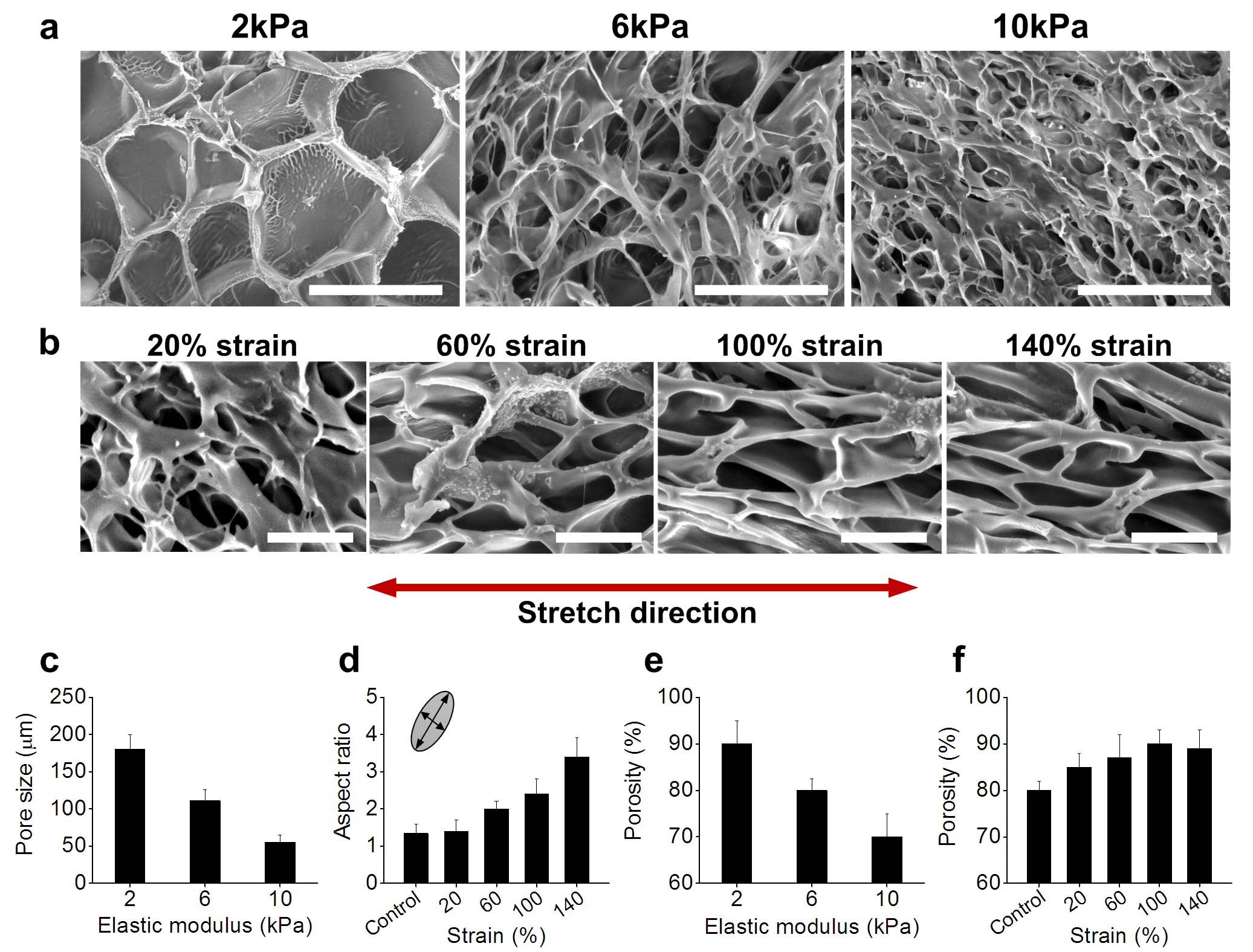
*

**Supplementary Figure 14** | Structural characterization of μMASTs. (**a**) SEM images of unstrained μMASTs (2 kPa, 6 kPa and 10 kPa). (**b**) Deformation of pore structure in 6 kPa μMASTs under varying strain levels. (**c**) Pore size of μMASTs decreased by increasing hydrogel modulus. (**d**) Aspect ratio of pores in 6kPa group under applied strain ranging from 20%-140%. (e-f) Porosity of μMASTs with varying modulus (e) and applied strains in 6 kPa group (f). Error bars, s.d. (n = 3 μMASTs for each bar). Scale bars: (a) 200 μm for 2 kPa, 100 μm for 6 kPa and 10 kPa, (b) 50 μm.


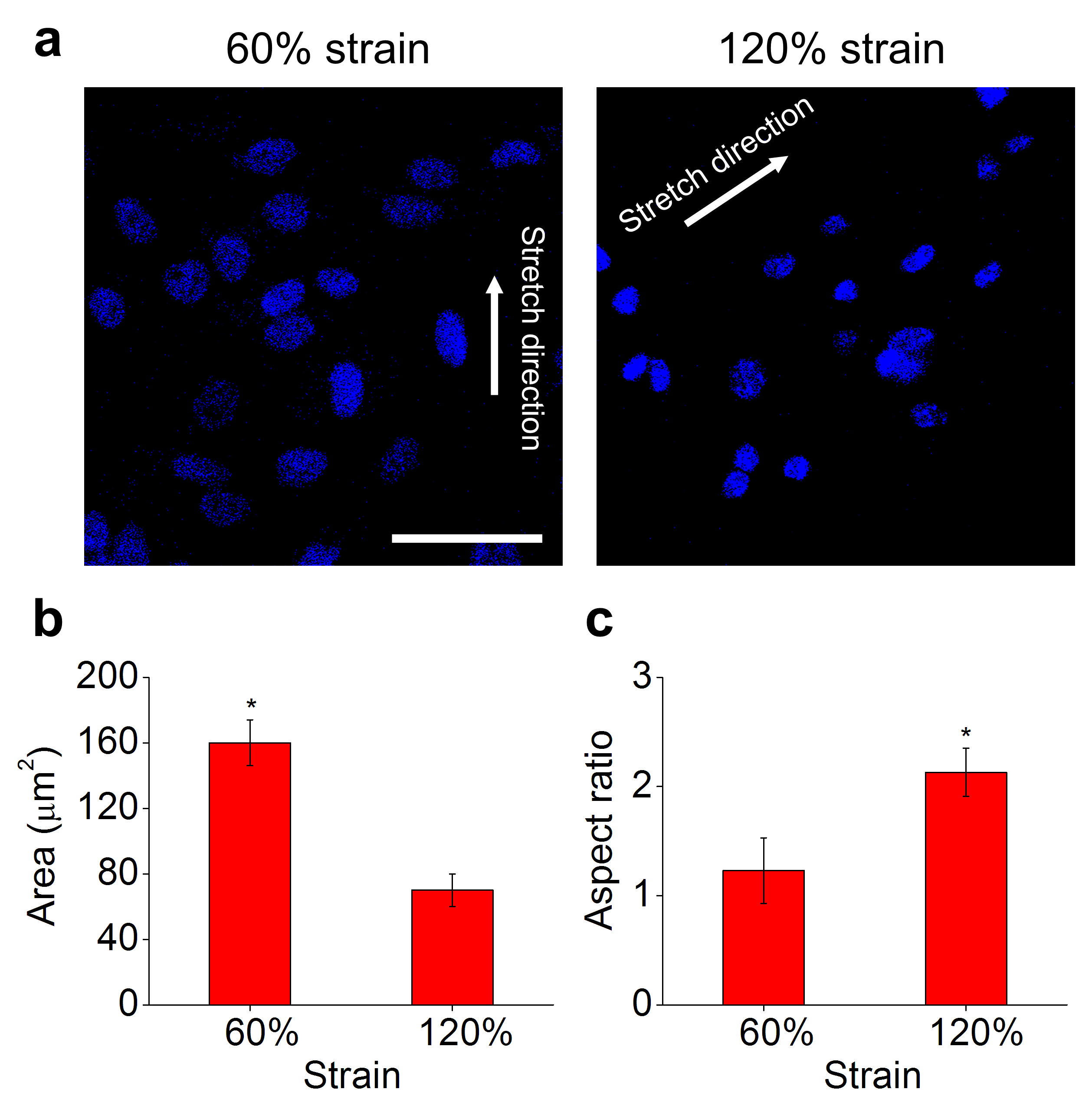


**Supplementary Figure 15** | Quantification of nuclear morphology. (**a**) Confocal florescent images of cell nucleus in 6 kPa synthetic tissues with 60% and 120% strain, respectively. (**b-c**) Quantification results of nuclear area and aspect ratio at day 5 of culturing. n=10 μMASTs, **p* < 0.05. Scale bar: 50 μm.

**
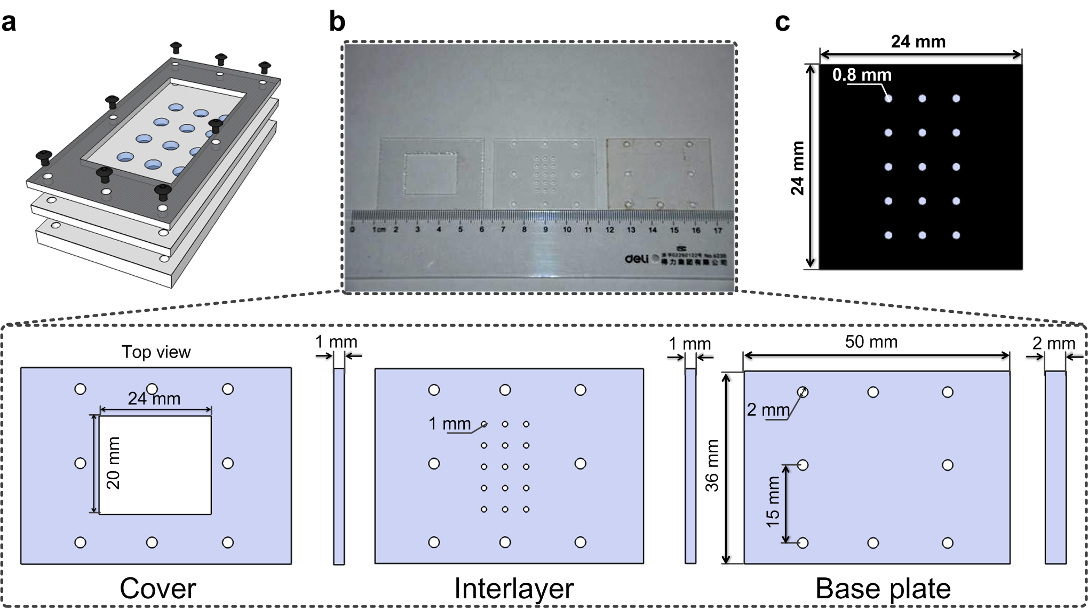
**

**Supplementary Figure 16** | PMMA mold and photomask. (**a**) The PMMA mold was composed of three parts: a cover, an interlayer, and a base plate. (**b**) Images and schematics of the three layers. (**c**) Schematic of the photomask. (The schematic was drawn by Yuhui Li using SketchUp Pro 8.0)

*
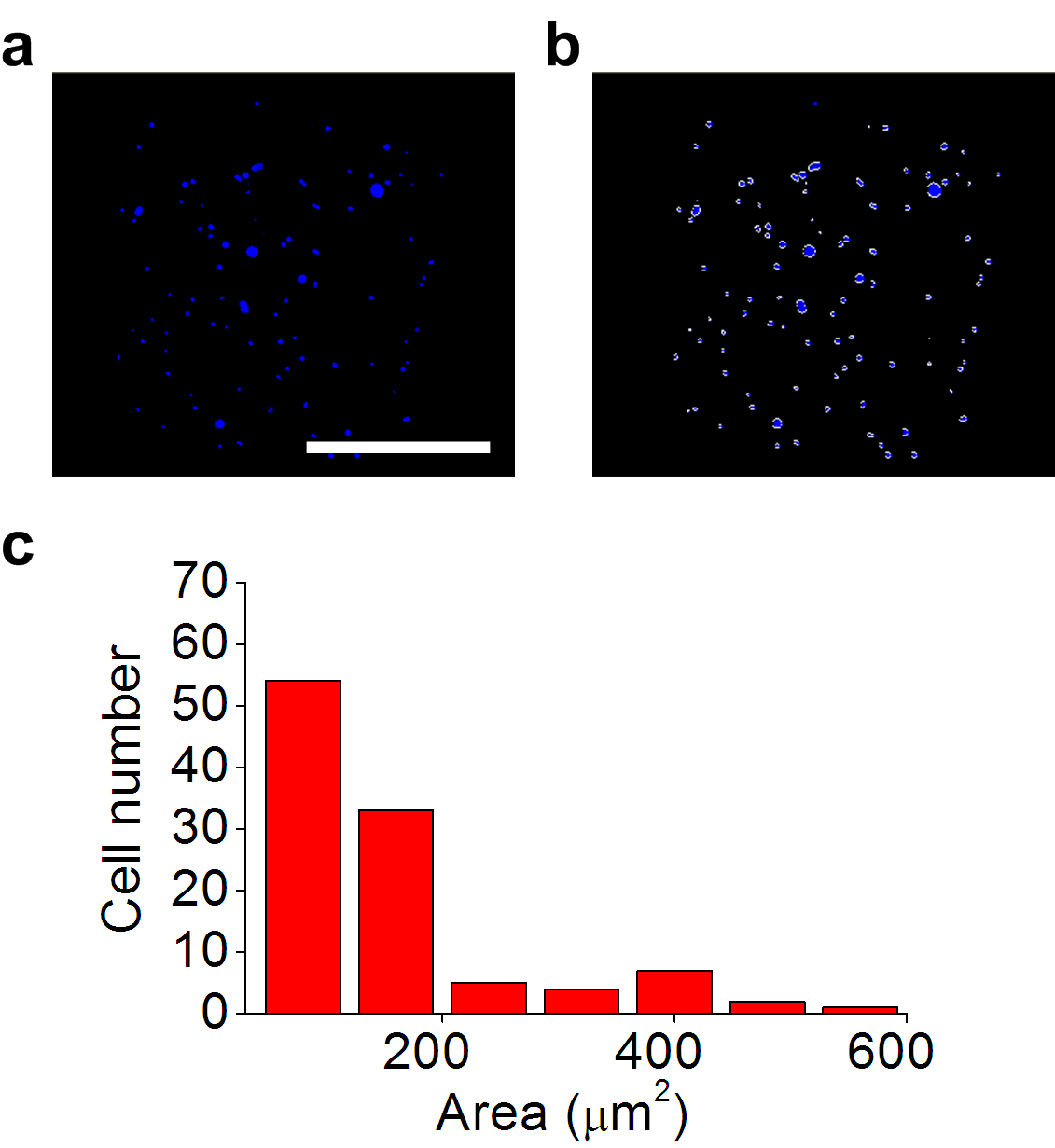
*

**Supplementary Figure 17** | Quantification methods of cell numbers in per μMAST. (a) Fluorescent images of cell nucleus in unstrained μMASTs after encapsulation. (b) Quantification image of nuclear number and area after “count/size-measure-select measurement-area” option in IPP. (c) Area distribution histogram of cell objects in fluorescent image. Scale bars: 500 μm.
